# High-resolution transcriptional analysis of the regulatory influence of cell-to-cell signalling reveals novel genes that contribute to *Xanthomonas* phytopathogenesis

**DOI:** 10.1111/mmi.12229

**Published:** 2013-04-26

**Authors:** Shi-Qi An, Melanie Febrer, Yvonne McCarthy, Dong-Jie Tang, Leah Clissold, Gemy Kaithakottil, David Swarbreck, Ji-Liang Tang, Jane Rogers, J Maxwell Dow, Robert P Ryan

**Affiliations:** 1 Division of Molecular Microbiology, College of Life Sciences, University of DundeeDundee, UK; 2 Division of Molecular Medicine, College of Life Sciences, University of DundeeDundee, UK; 3 Department of Microbiology, Biosciences Institute, University College CorkCork, Ireland; 4 State Key Laboratory for Conservation and Utilization of Subtropical Agro-bioresources, College of Life Science and Technology, Guangxi UniversityNanning, Guangxi, China; 5 The Genome Analysis CentreNorwich Research Park, Colney Lane, Norwich, NR4 7UH, UK

## Abstract

The bacterium *Xanthomonas campestris* is an economically important pathogen of many crop species and a model for the study of bacterial phytopathogenesis. In *X. campestris*, a regulatory system mediated by the signal molecule DSF controls virulence to plants. The synthesis and recognition of the DSF signal depends upon different Rpf proteins. DSF signal generation requires RpfF whereas signal perception and transduction depends upon a system comprising the sensor RpfC and regulator RpfG. Here we have addressed the action and role of Rpf/DSF signalling in phytopathogenesis by high-resolution transcriptional analysis coupled to functional genomics. We detected transcripts for many genes that were unidentified by previous computational analysis of the genome sequence. Novel transcribed regions included intergenic transcripts predicted as coding or non-coding as well as those that were antisense to coding sequences. In total, mutation of *rpfF*, *rpfG* and *rpfC* led to alteration in transcript levels (more than fourfold) of approximately 480 genes. The regulatory influence of RpfF and RpfC demonstrated considerable overlap. Contrary to expectation, the regulatory influence of RpfC and RpfG had limited overlap, indicating complexities of the Rpf signalling system. Importantly, functional analysis revealed over 160 new virulence factors within the group of Rpf-regulated genes.

## Introduction

Many bacteria use cell-to-cell communication mediated by diffusible signal molecules to monitor aspects of their environment and modulate their behaviour accordingly. Cell-to-cell signalling allows a colony or group of organisms to act in a co-ordinated fashion to regulate diverse processes such as the formation of biofilms, synthesis of antibiotics and the production of virulence factors in pathogenic bacteria (Whitehead *et al*., 2001; Lyon and Novick, 2004; Waters and Bassler, 2005; Konaklieva and Plotkin, 2006). The signal molecules synthesized by bacteria belong to a wide range of chemical classes and a diverse range of mechanisms for signal perception and transduction has been described (Diggle *et al*., 2006; Higgins *et al*., 2007). For many bacteria, comparative transcriptional profiling has been used to extend our understanding of these various signalling mechanisms and of the processes that are controlled (Raskin *et al*., 2006). This knowledge may suggest strategies for interference with consequences for disease control. In recent years, our appreciation of the complexity and regulation of bacterial transcriptomes has been revised through the use of RNA sequencing (RNA-Seq) (recently reviewed by Croucher and Thomson, 2010; Febrer *et al*., 2011; Maeder *et al*., 2011). The use of such next-generation technologies offers a deeper insight into the molecular mechanisms of bacterial signalling and of the regulated processes.

Bacteria belonging to the genus *Xanthomonas* cause diseases in many economically important plants throughout the world (Ryan *et al*., 2011; Mansfield *et al*., 2012). These phytopathogens use cell-to-cell signalling mediated by molecules of the Diffusible Signal Factor (DSF) family to regulate expression of factors contributing to virulence (Ryan *et al*., 2011). The DSF family of signals are *cis*-2-unsaturated fatty acids, for which the paradigm is *cis*-11-methyl-dodecenoic acid described from the crucifer pathogen *Xanthomonas campestris* pv. *campestris* (*Xcc*) (reviewed by Ryan and Dow, 2011). DSF signalling in *Xcc* has been shown to influence the synthesis of virulence factors such as extracellular enzymes and extracellular polysaccharides (Ryan and Dow, 2011). Although elements involved in DSF signal transduction have been described, much remains to be understood about their actions and the role of DSF signalling in phytopathogenesis.

Work in *Xcc* has demonstrated that both the synthesis and perception of the DSF signal require products of the *rpf* gene cluster (for regulation of pathogenicity factors). The synthesis of DSF is dependent on RpfF, which belongs to the crotonase superfamily of enzymes, whereas the two-component system comprising the sensor kinase RpfC and regulator RpfG is implicated in DSF perception and signal transduction (Slater *et al*., 2000; Ryan *et al*., 2006; Ryan and Dow, 2008). RpfC is a complex hybrid sensor kinase whereas the RpfG regulator has a CheY-like receiver (REC) domain attached to an HD-GYP domain, which acts to degrade the second messenger bis (3′, 5′)-cyclic diguanosine monophosphate (cyclic di-GMP) (Ryan *et al*., 2006; 2010). Mutation of *rpfF*, *rpfG*, or *rpfC* in *Xcc* leads to a co-ordinate reduction in the synthesis of virulence factors such as the extracellular enzymes protease, endoglucanase, and endomannanase and the extracellular polysaccharide (EPS) xanthan, alterations in biofilm formation and a reduction in virulence (Slater *et al*., 2000; Dow *et al*., 2003; Ryan *et al*., 2006). Addition of DSF can restore virulence factor synthesis to *rpfF* mutants but not to *Xcc* strains with mutations in *rpfG* or *rpfC*, consistent with the involvement of RpfC/RpfG in perception and transduction of the DSF signal (Slater *et al*., 2000; Ryan *et al*., 2006). However, mutation of *rpfC* and *rpfG* has opposite effects on DSF synthesis, suggesting the existence of additional complexity in the Rpf/DSF regulatory system. RpfG and RpfC are encoded as part of the *rpfGHC* operon (Slater *et al*., 2000). RpfH is a predicted membrane-associated protein with amino acid sequence similarity to the sensory input domain of RpfC, but with no known role in DSF signalling or virulence (Slater *et al*., 2000).

The work in this article had the aim of increasing our understanding of the actions of the individual Rpf components and of the overall regulatory influence of the Rpf/DSF signalling system on phytopathogenesis of *Xcc*. Here we describe the use of RNA-Seq to compare the transcriptomes of wild-type and isogenic *rpf* mutants in a comprehensive fashion. This analysis has allowed us to identify many genes that were unidentified by previous computational analysis of the genome sequence and novel transcribed regions of the genome (for example, identification of potential non-coding RNA (ncRNA) genes) that are under Rpf/DSF control. Importantly mutational studies allowed us to describe over 160 new virulence factors for *Xcc* within the group of genes under the control of the Rpf/DSF system; these factors comprised over 140 previously annotated genes, 16 of these ‘new’ genes and three novel ncRNAs.

## Results

### The transcriptome structure of *Xcc* 8004

In this study we used RNA-Seq to characterize at high-resolution the global gene expression pattern of *Xcc* when grown in complex medium. Total RNA was isolated from planktonic cultures of *Xcc* 8004 growing in exponential phase. Three biological replicates (each comprising three technical replicates for a total of nine samples) were analysed (see *Experimental procedures* for specific details). After depletion of rRNA by the Gram-negative Ribo-Zero™ kit (Epicentre), barcoded cDNA libraries were generated from the RNA-samples. All samples were sequenced on flowcell lanes of an Illumina HiSeq2000. The raw sequence output consisted of approximately 50 million reads per sample, each with a length of 50–100 nucleotides (see Table S1). Non-ribosomal reads that did not align uniquely with the genomic sequence were discarded.

The primary annotation of the *Xcc* 8004 genome comprises 4271 genes, including 2671 genes that encode proteins with functional assignment and 53 that encode structural RNAs (Qian *et al*., 2005). A number of reads, including some of high abundance, were found to unambiguously match the *Xcc* 8004 genome sequence at locations that were not previously annotated as genes (e.g. in intergenic regions) or that were overlapping with annotated ORFs.

To further examine genes that lay outside of the primary annotation and retain them as evidence for *bona fide* new transcripts, three previously defined criteria (Beaume *et al*., 2010; Legendre *et al*., 2010) were applied: (i) the beginning of the new transcript had to be defined by at least five overlapping reads exhibiting the 5′ end tag, (ii) the end of the transcript had to be defined by at least five overlapping reads exhibiting the 3′ end tag, and (iii) the new transcript delimited by these 5′ and 3′ boundaries had to correspond to an uninterrupted tiling of contiguous reads. Using these constraints, manual annotation identified 1190 transcriptional units (genes) that were re-defined in the *Xcc* 8004 NC_007086 genome while an additional 321 previously unannotated genes were defined. These new transcripts were mostly located within intergenic regions significantly larger than the average (which for *Xcc* is 100 nt, *P* < 10^−7^), thus filling up genome segments of previously lower gene density. On the basis of coding potential (see *Experimental procedures*), 181 of these new transcripts were classified as putative protein-coding genes. blast analysis identified that 30 of the CDSs have been identified and annotated in other xanthomonad genomes, whereas the remaining 151 appeared to be novel. Conserved domains were identified in a number of these novel proteins (Table S2). It is important to note that our approach can only detect termini of bacterial transcripts with certainty but cannot unambiguously assign transcriptional start sites due to lack of strand discrimination. However, recent studies using a stand-specific approach (dRNA-Seq) have generated a comprehensive transcription start site map in a related Xanthomonad species *X. campestris* pv. *vesicatoria* (*Xcv*) (Schmidtke *et al*., 2011). By cross-referencing the data from this study we have been able to validate 1024 transcription start sites in *Xcc*.

**Fig. 1:**
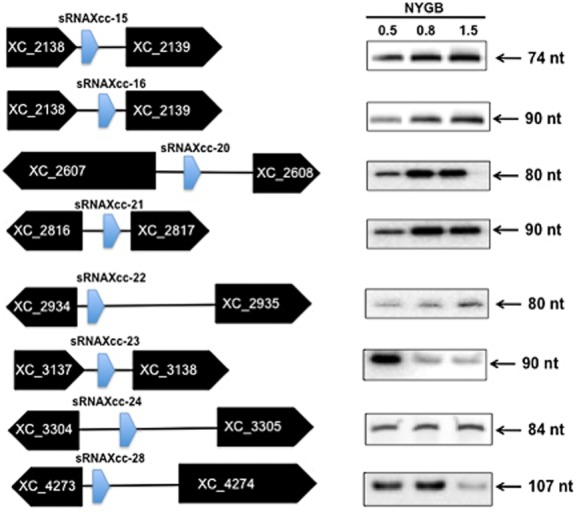
Northern blot analysis of selected small non-coding RNAs candidates from *Xcc*. Schematic diagrams of the genomic context of each sRNA are given on the left. Northern hybridizations were carried out on RNA samples isolated from wild-type cells cultured in NYGB medium. The optical density at 600 nm of the cultures is indicated. The probes used in Northern analysis were complementary to the sRNA. Arrows indicate the predominant bands. Sizes of the sRNAs are given on the right. rRNAs served as control for RNA loading and RNA integrity. A summary of information on sRNAs verified is detailed in Table S4.

To identify possible ncRNAs, the 1181 intergenic regions were analysed *in silico* using an adaptation of the approach of Mandin *et al*. (2007) as detailed in *Experimental procedures*. This analysis identified 24 transcripts as putative ncRNAs (length = 75 nt ± 25 nt). The regions representing these putative ncRNAs showed contiguous coverage by RNA-Seq reads (i.e. at least 100 bp completely covered by RNA-Seq reads). All of these putative ncRNAs identified here were not previously identified in other *Xanthomonas* strains and none matched ncRNA entries in Rfam (see Table S3). The eight most promising candidates for novel ncRNAs as judged by the use of Mfold were validated by Northern hybridization which showed the presence of a transcript of the appropriate size (Fig. 1).

In parallel to the analysis described above, sequence reads were mapped to the *Xcc* 8004 NC_007086 reference genome using both the RAST (Rapid Annotation using Subsystem Technology) (Aziz *et al*., 2008) and Prodigal (Prokaryotic Dynamic Programming Gene-finding Algorithm) (Hyatt *et al*., 2010) systems as detailed in *Experimental procedures*. Overall, analysis with Prodigal detected 126 genes that lay outside of the primary annotation of *Xcc* 8004 of which 101 were in entirely intergenic regions. While, RAST software identified 379 genes that lay outside of the primary annotation with 261 being intergenic. Thus all methods of analysis revealed previously unidentified genes. For the purpose of clarity, the manual analysis described above will be used to define the wild-type transcriptome in the comparative transcriptomic analyses described in the following sections.

In summary, transcripts belonging to the following five categories were found: previous annotated CDSs; re-defined CDSs; antisense to established CDSs and intergenic transcripts predicted as coding or non-coding. This establishes the total gene number at 4374 (including 24 ncRNAs + 181 new transcripts). Under the growth conditions used, the transcribed fraction of the *Xcc* 8004 NC_007086 genome is 98%. We do not rule out that additional transcripts (and hence new genes) may be discovered under different conditions or in different phases of growth. A comprehensive graphical transcriptome map of *Xanthomonas* was generated and is available at XanthomonasGbrowse. This map provides a high-resolution view of the *Xanthomonas* transcriptome structure.

### Influence of the Rpf/DSF signalling system on the *Xcc* transcriptome

In order to examine the regulatory influence of the Rpf/DSF signalling system, the impact of mutation of *rpfF, rpfC, rpfH* and *rpfG* on the *Xcc* transcriptome was established by RNA-Seq. An average total of 53 million reads was acquired for each mutant sample. The numbers of the reads from each pooled mutant sample that were used for further analysis after trimming are detailed in Table S1. There was only limited variability between the total number of reads for the different strains tested. For each pooled sample, alignments to the *Xcc* 8004 NC_007086 reference genome were generated; the total of mapped reads and a total of unmapped reads are detailed in Table S1. All of these unmapped reads were identified within the group of 181 new protein-encoding transcripts found in our analysis of the wild-type *Xcc* 8004 genome.

The numbers of reads were used to calculate the normalized gene expression as Fragments Per Kilobase per Million mapped fragments (FPKM). The differential gene expression of the pooled samples from wild-type and mutants were analysed using the R sequence package Cufflinks (Trapnell *et al*., 2010; 2012) (see *Experimental procedures*). Differentially expressed genes with a fold change ≥ 4.0 and a stringent *P*-value of less than 0.001 along with their assigned annotations are listed for each mutant in Table S4. These regulated genes collectively were involved in a range of biological functions including virulence, membrane transport, multidrug resistance, amino acid biosynthesis and signal transduction.

**Fig. 2:**
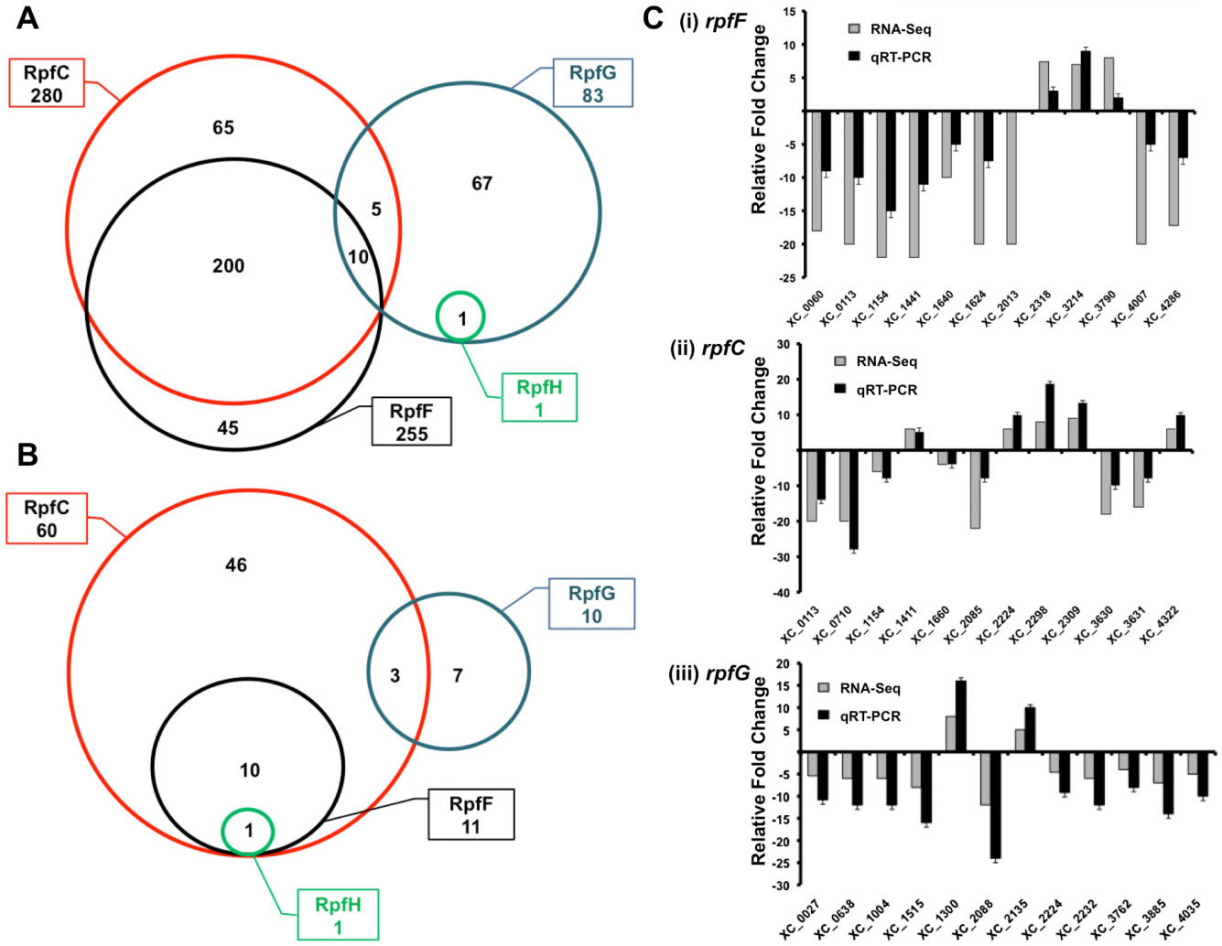
Changes in gene expression of *rpfF*, *rpfC*, *rpfG* and *rpfH* mutants compared with the wild-type *Xanthomonas campestris* 8004 as measured by RNA-Seq. A and B. Venn diagrams showing the overlap of genes whose expression is (A) upregulated or (B) downregulated in different mutant backgrounds. Divergently regulated genes are not depicted in these Venn diagrams but can be found in Table S5. C. Comparison of relative fold changes between RNA-Seq and qRT-PCR results in (i) *rpfF*, (ii) *rpfC* and (iii) *rpfG* mutant backgrounds. All qRT-PCR results were normalized using the *Ct*s obtained for the 16S rRNA amplifications run in the same plate. The relative levels of gene transcripts are determined from standard curves. Values given are the mean and standard deviation of triplicate measurements (three biological and three technical replicates).

This transcriptome profile analysis revealed that expression of 272 genes was significantly altered by the inactivation of *rpfF*. Deletion of *rpfC* appeared to have a broader influence on global gene expression as 359 genes showed significantly altered expression in the mutant compared with wild-type (Fig. 2). In contrast, analysis of the *rpfG* mutant revealed 112 genes that were differentially expressed. Notably, the deletion of *rpfH* had a minor effect on the *Xcc* transcriptome with alteration of expression of only 4 genes. Importantly, known *Xcc* 8004 reference or housekeeping genes such as *gyrB*, *proC*, *recA*, *atpD* and *dnaK* were not differentially expressed between the wild-type and mutant samples.

Given that our previous work in *Xcc* has demonstrated that both the synthesis and perception of the DSF signal require products of the *rpf* gene cluster, it is not unexpected that overlap in the regulatory influence of each *rpf* gene was seen (Fig. 2). Mutation of *rpfF*, *rpfC* and *rpfG* led to the altered expression of a common set of genes (see Fig. 2). Reassuringly, this set of genes included those encoding endoglucanase, various proteases and endomannanase, previously reported to be co-ordinately regulated by the different elements within the Rpf/DSF signalling system (Slater *et al*., 2000). Contrary to expectations, mutation of *rpfC* and *rpfG* (encoding the sensor and regulator of the RpfGC two-component system) only demonstrated a limited regulatory overlap on gene expression using the specific cuts-offs we employed; a total of 36 genes were affected by both RpfG and RpfC, 19 of which were divergently regulated. Furthermore, this transcriptional analysis revealed that RpfF, RpfC and RpfG had considerable independent effects on gene transcription (Fig. 2; Table S5).

In addition examination of the eight validated ncRNAs demonstrated that three of these (sRNAXcc-15, sRNAXcc-16 and sRNAXcc-28) were differentially regulated by the Rpf/DSF system, being influenced by RpfC and RpfF but not by RpfG.

Quantitative RT-PCR methods were used to confirm alterations in gene expression revealed by RNA-Seq. The genes selected for these analyses represented those with a range of fold change of expression and of diverse functional classes. The relative expression levels of 40 genes using qRT-PCR in each mutant measured reflected the differences in gene expression observed by transcriptome analysis (Fig. 2; Table S6). Taken together, the findings, which we will discuss below, indicate a hitherto unsuspected level of complexity within the Rpf/DSF regulatory system.

### Functional genomic assessment of regulated genes identifies novel factors involved in phytopathogenesis

Since mutation of *rpfF*, *rpfG* or *rpfC* all lead to a reduction in virulence of *Xcc*, it follows that some of the elements identified above to be Rpf-regulated may have a role in virulence or virulence factor synthesis. In order to examine this, the effects of mutation of 480 annotated genes collectively regulated by the Rpf/DSF system on virulence to Chinese radish were assessed (see *Experimental procedures*). To our knowledge, approximately 15 of these genes have been implicated previously in virulence. Mutants with single Tn*5gusA* insertions in genes of interest were identified within a library of such mutants (see *Experimental procedures*). Mutations created using the Tn*5gusA* transposon may have polar effects on downstream genes in the same transcriptional unit, complicating the interpretation of the phenotypes. Some of these polarity problems were overcome by the use of pK18*mob* derivatives for insertional inactivation, in which an outward-facing promoter in the vector will drive the expression of downstream genes. In other cases, the phenotypic effects of Tn*5gusA* insertion in downstream genes were also tested. For selected genes, the polarity problem was avoided by the creation of in-frame deletions (see *Experimental procedures*).

**Fig. 3:**
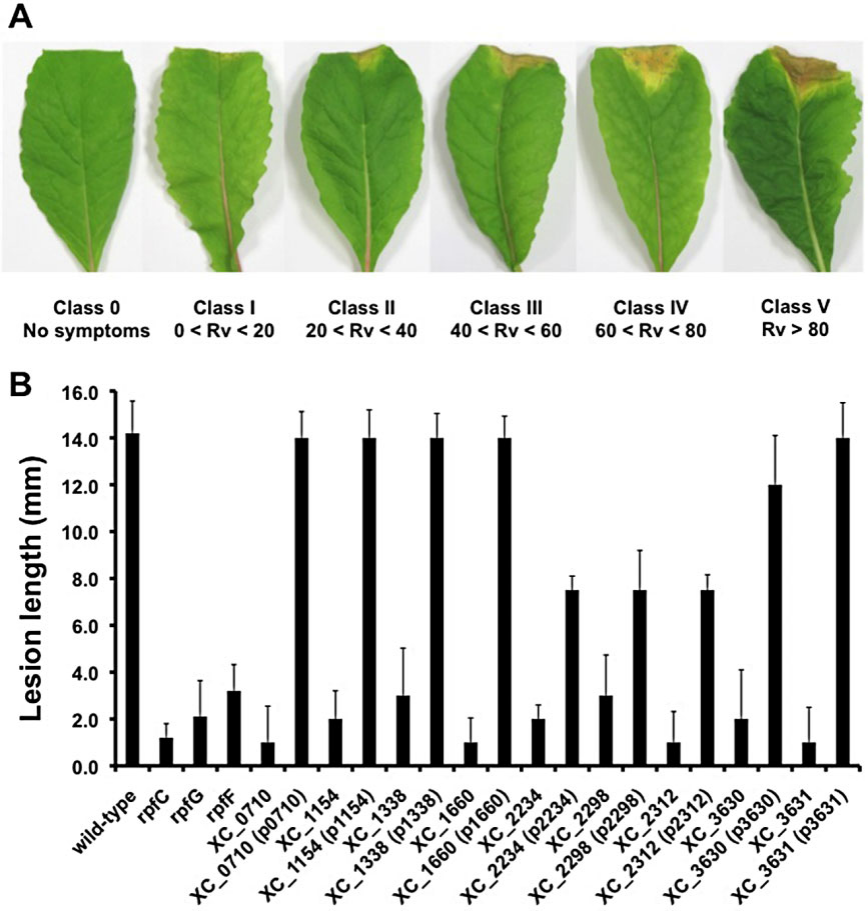
Reduced virulence phenotypes in Chinese radish as a consequence of mutation of genes regulated by the Rpf/DSF signalling system. A. The virulence of each mutant was tested by measurement of the lesion length after bacteria were introduced into the vascular system of Chinese radish by leaf clipping as detailed in the *Experimental procedures*. Mutants were assigned to classes I–V depending on the percentage lesion length when compared with the *Xcc* wild-type strain (Rv). B. The complementation of mutants that were in Class I. The virulence of each mutant and complemented strain was tested by measurement of the lesion length after bacteria were introduced into the vascular system of Chinese radish by leaf clipping.

*Xcc* is a vascular pathogen and is normally restricted to the xylem of leaves of infected plants at the early stage of disease. The virulence of each mutant was tested by measurement of the lesion length after bacteria were introduced into the vascular system of Chinese radish by leaf clipping (Ryan *et al*., 2007; 2010). These strains were tested for virulence on four sets of 30 leaves for each mutant. The virulence phenotype in Chinese radish was classed according to categories illustrated in Fig. 3.

Mutation of 148 previously annotated genes gave a significant reduction in virulence in repeated tests (Table S7). The most profound effects on virulence were seen with mutation of *XC_0710, XC_1038, XC_1154, XC_1660, XC_2313, XC_2234, XC_2298, XC_3630* and *XC_3631*. Except in the case of two genes that encode hypothetical proteins, the products of these genes were associated with known or predicted functions such as flagellar biogenesis, chemotaxis and signalling. None of these genes had been previously associated with *Xanthomonas* virulence. Importantly, virulence was restored towards wild-type by complementation *in trans* with a wild-type copy of the appropriate gene (Fig. 3).

No mutation lead to a complete loss of virulence in Chinese radish. To ascertain if the effects of any of the mutations on virulence were additive, double mutants were created in strains that showed virulence phenotypes in Classes II or III. A complete list of double mutants tested is detailed in Table S8. These strains were tested for virulence on four sets of 30 leaves for each mutant. There was however no apparent additive effect of stacking mutations.

This analysis was extended to ‘newly revealed’ genes and the three ncRNAs regulated by the Rpf/DSF system. Sixty-eight of the 181 novel CDSs that were identified were regulated by the Rpf/DSF system (Table S9). Mutations were created in these candidates using the transposon and insertional inactivation strategies as detailed above (see *Experimental procedures*) and the panel of mutants was tested *in planta*. Mutation of 16 genes gave a significant reduction in virulence in repeated tests compared with wild-type (Table 1; Table S9). The majority of these genes encoded hypothetical proteins (Table 1).

**Table 1:** Mutation of previously unannotated genes regulated by RpfF, RpfC and/or RpfG that influence virulence of *Xcc* to Chinese radish

Designation^a^	Start^b^	End^b^	Length (nt)	Predicted function^c^	Homologues in other organisms^c^	Average lesion length (mm)^d^	Virulence class^d^
TID2	50022	50402	381	Hypothetical protein	Xcc, Xca, Xcr	0.0 ± 0.0	I
TID5	80767	81213	447	Hypothetical protein	Xcc, Xcr, Xoo, Xcv, Xca, Ps	5.0 ± 0.8	II
TID28	577673	577957	285	Hypothetical protein	Xcc, Xcr, Xoo, Xcv, Xca, Ps, Steno	10.1 ± 0.3	IV
TID32	710291	710434	144	Hypothetical protein	Xcc, Xcr, Xac	9.4 ± 0.3	IV
TID36	838746	839714	969	Conjugation protein TraG	Xcc, Xcr, Xoo, Xcv, Xca, Ps, Steno, Burk	1.8 ± 0.0	I
TID60	1265318	1265524	207	Hypothetical protein	Xcc, Xcr, Xoo, Xcv, Xca, Ps, Steno, Burk	3.3 ± 0.7	II
TID64	1568785	1569600	816	Hypothetical protein	Xcc, Xcr, Xac	3.9 ± 0.8	II
TID68	1744147	1744494	348	Hypothetical protein	Xcc, Xcr, Xac	0.5 ± 0.1	I
TID75	2193981	2194241	261	Hypothetical protein	Xcc, Xcr, Xac	9.5 ± 0.8	IV
TID85	2611087	2611344	258	Stress protein	Xcc, Xcr, Xoo, Xeu, Xca, Ps, Steno, Denio, Herba, Methylo	10.1 ± 0.3	IV
TID118	3140515	3140955	441	Hypothetical protein	Xcc, Xcr, Xoo, Deino, Ral, Sor	4.1 ± 0.2	II
TID120	3148912	3149193	282	Hypothetical protein	Xcc, Xcr, Xoo, Deino, Ral, Sor	1.5 ± 0.1	I
TID135	3627629	3627952	324	Dioxygenase	Xcc	5.2 ± 0.3	II
TID153	4305065	4305484	420	Transposase	Xcc, Xca, Xoo, Nitro, Rhizo, Methylo, Aceto, Brady, Beij	10.7 ± 0.7	IV
TID155	4413491	4414270	780	Hypothetical protein	Xcc, Xca, Xoo, Nitro, Rhizo, Methylo, Aceto, Brady, Beij	7.6 ± 0.7	III
TID177	5068515	5068715	201	Hypothetical protein	Xcc, Xca, Xoo, Nitro, Rhizo, Methylo, Aceto, Brady, Beij	5.1 ± 0.4	II

a. Designation of novel transcripts adapted from Table S2.

b. Annotation taken from Qian *et al*. (2005).

c. Best blast hits searching sequenced bacterial genomes. *Xanthomonas campestris* pv. *campestris* (Xcc) *Xanthomonas citri* (Xca); *Xanthomonas campestris* pv. *raphani* (Xcr); *Xanthomonas oryzae* pv. *oryzae* (Xoo); *Xanthomonas oryzae* pv. *oryzicola* (Xoc); *Xanthomonas euvesicatoria* (Xeu); *Stenotrophomonas* sp. (Steno); *Pseudomonas* sp. (Ps); *Ralstonia* sp. (Ral); *Burkholderia* sp. (Burk); *Deinococcus* sp. (Denio); *Herbaspirillum* sp. (Herba); *Acetobacter* sp. (Aceto); *Nitrobacter* sp. (Nitro); *Methylobacterium* sp. (Methylo); *Bradyrhizobium* sp. (Brady); *Beijerinckia* sp. (Beij).

d. Reduced virulence phenotypes in Chinese radish as a consequence of mutation of genes as adapted from classification defined in Fig. 3.

The virulence of each mutant was tested by measurement of the lesion length after bacteria were introduced into the vascular system of Chinese radish by leaf clipping.

**Fig. 4:**
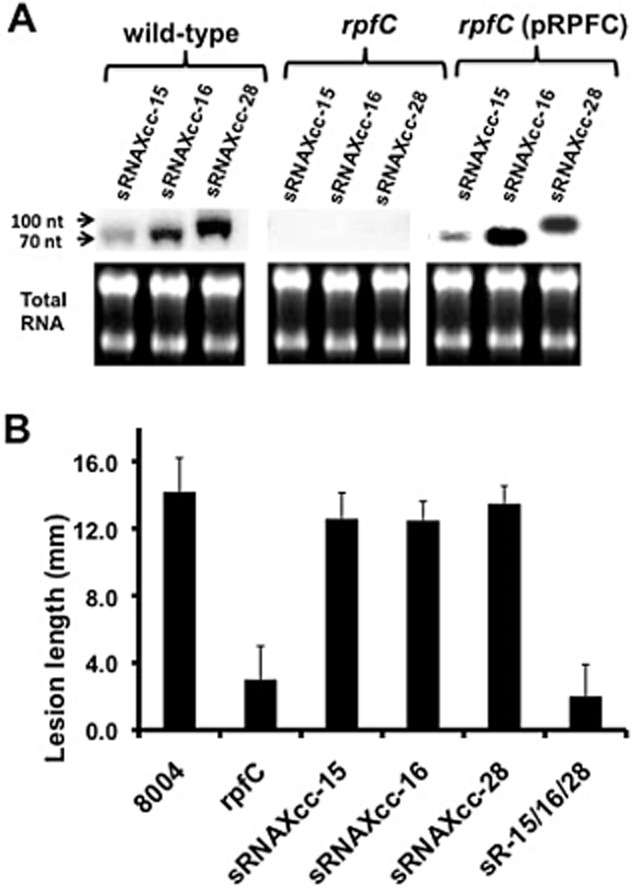
Regulation of expression of three sRNAs by the Rpf/DSF system and their involvement in virulence. A. Northern blots of the selected sRNAs (sRNAXcc-15, sRNAXcc-16, sRNAXcc-28) regulated by the Rpf/DSF system. RNA isolated from *Xcc* 8004 wild-type (i), *rpfC* mutant (ii) and complemented *rpfC* mutant [*rpfC* (pRPFC)] (iii) was probed for sRNAs. B. A triple deletion of sRNAXcc-15/sRNAXcc-16/sRNAXcc-28 (sR15/16/28) leads to reduction in virulence as measured in Chinese radish by leaf clipping. In contrast deletion of sRNAXcc-15, sRNAXcc-16, sRNAXcc-28 alone had no effect on virulence.

To assess the contribution of the three ncRNAs (sRNAXcc-15, sRNAXcc-16 and sRNAXcc-28) under the control of the Rpf/DSF system to virulence, we generated a deletion mutant in each candidate. No difference in the ability to cause disease was seen between each single-deletion mutant and the wild-type strain (Fig. 4). As a considerable level of redundancy has been observed in ncRNA regulation in other bacterial systems, we were prompted to examine the virulence of a mutant carrying a deletion of all three ncRNA genes (strain sR-15/16/28). Deletion of all three ncRNAs showed a considerable reduction in virulence (Fig. 4).

Overall this analysis has revealed a significant number (over 150) of novel virulence determinants for *Xcc*. This subset of new virulence factors for *Xcc* includes a number of hypothetical proteins that are uncharacterized and lack conserved domains. Interestingly, many of these proteins have homologues in other xanthomonads, including other *Xanthomonas* species and *Stenotrophomonas*. Other new virulence factors with conserved domains also have homologues in other xanthomonads, as well as in other genera such as *Ralstonia* and *Pseudomonas;* nine of these are widely distributed in bacteria (Table 1; Table S7).

## Discussion

Although the significance of Rpf/DSF signalling for the virulence of many *Xanthomonas* spp. is now well established, much remains to be understood about the actions of the individual Rpf components and how they influence phytopathogenesis. The work in this article had the aim of using RNA-Seq to define the regulatory influence of different Rpf proteins on the transcriptome of *Xcc*.

The ability to reliably identify differentially expressed genes by RNA-Seq is affected by a variety of factors (Tarazona *et al*., 2011). Recently, Haas and colleagues conducted a systematic examination of the impact of depth of sequencing, rRNA removal and pooling of samples on single bacterial transcriptome profiling and gene expression assessment (Haas *et al*., 2012). This study suggested a set of strong guidelines for seeking optimum sequencing depth and conditions for RNA-Seq studies of bacteria. The parameters of the method applied here fall within the guidelines set by Haas *et al*. (2012); for example to avoid spurious transcript identification events optimal sequencing coverage should be between 10 million and 75 million fragments per sample.

This approach has allowed us to identify many genes that were unidentified by previous computational analysis of the *Xcc* genome sequence and to provide insight into the distinct functions of the different Rpf proteins. Furthermore, functional genomic analyses directed by the knowledge gained from comparative transcriptomics has allowed us to identify a large suite of new factors contributing to *Xcc* virulence. Notably, many of these new factors are conserved in other phytopathogens.

### Complexities of the Rpf signalling system

The *rpf* genes were first identified in *Xcc* through their influence on the production of specific extracellular enzymes such as endoglucanase and protease. The phenotypes of different *rpf* mutants for extracellular enzyme production and effects of addition of DSF are consistent with the involvement of RpfC/RpfG in perception and transduction of the DSF signal in a linear pathway leading to regulation of extracellular enzyme production. The findings reported here support these earlier conclusions in that mutation of *rpfF*, *rpfC* and *rpfG* all give rise to reduced transcript levels for genes encoding endoglucanase, various proteases and endomannanase. Nevertheless, the genes influenced co-ordinately by *rpfF*, *rpfC* and *rpfG* only represent a small proportion (∼ 2%) of those whose transcript level is significantly altered.

**Fig. 5:**
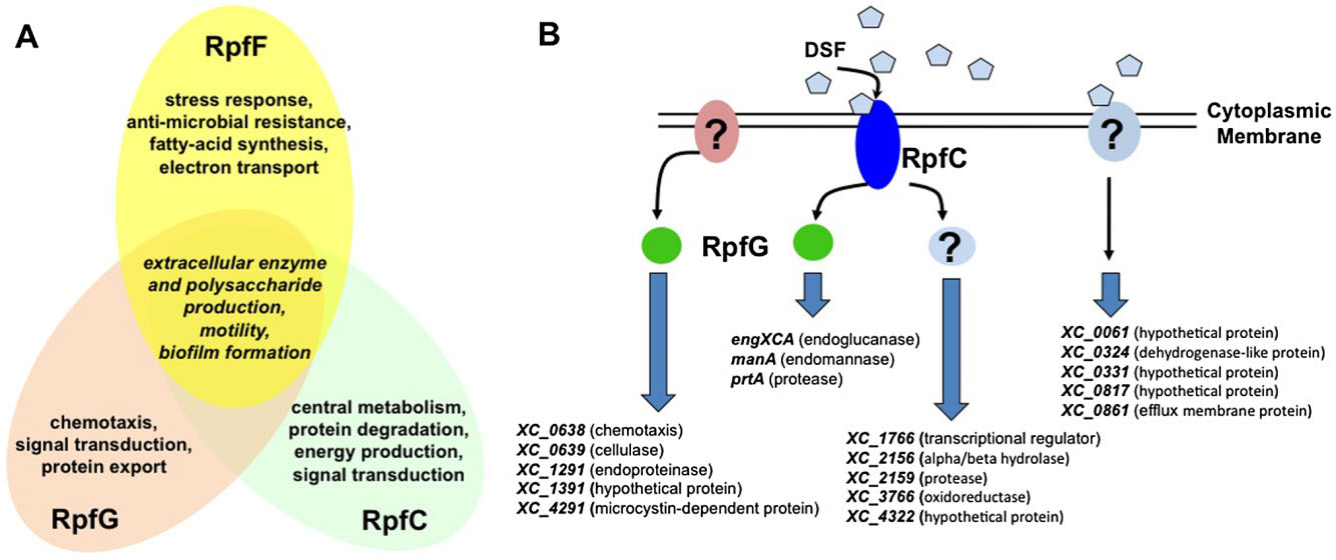
Model of the DSF/Rpf signalling cascades in *Xcc*. A. Venn diagram depicting the overlapping and discrete regulatory actions of RpfF, RpfG and RpfC that are involved in phytopathogenesis. B. Schematic representation of regulatory pathways involving DSF, RpfC and RpfG in the virulence of *Xcc*. Expression of several genes including *engXCA*, *prtA* and *manA* are co-ordinately regulated by RpfF, which synthesizes DSF and the RpfCG two-component system, consistent with the linear pathway previously described. However RpfG controls a number of genes including *XC_0638* (chemotaxis) that are not influenced by RpfF or RpfC, suggesting an interaction of RpfG with a second unknown sensor (left). DSF and RpfC also regulate expression of a number of genes including *XC_1766* (transcription regulator) in a pathway independent of RpfG (centre). RpfF appears to control a number of genes independently of the other Rpf proteins, suggesting several genes including *XC_0061* (hypothetical protein) are regulated by DSF but not by RpfG or RpfC. This is evidence for the occurrence of a second sensor and signal transduction system for DSF (right). All of the target genes indicated encode virulence factors that are novel, with the exception of *engXCA*, *prtA* and *manA*, whose role in virulence has been described previously. All predicted protein functions are given in parentheses.

Comparison of the regulatory influence of RpfF and RpfC demonstrate considerable overlap under the conditions tested. The transcript levels of 224 genes are commonly reduced in both the *rpfC* mutant (359 genes altered) and *rpfF* mutant (272 genes altered). Similarly all 17 genes with increased transcript level in the *rpfF* mutant are also increased in the *rpfC* mutant. These findings are consistent with the contention that transduction of the DSF signal requires RpfC. Nevertheless, RpfF regulates transcript levels of 48 genes that are not regulated by RpfC and conversely RpfC regulates transcript levels of 135 genes that are not regulated by RpfF. These findings suggest that RpfC can recognize other environmental signals (in addition to DSF) and point to the possibility that additional mechanisms for DSF perception leading to alteration in gene expression exist in *Xcc* (Fig. 5).

Contrary to expectation, the regulatory effects of RpfC and RpfG on transcript levels have limited overlap. Furthermore mutations of *rpfG* and *rpfC* have divergent effects on transcript levels of a number of genes. These observations suggest the existence of other regulators that interact with RpfC to effect these changes in gene expression. Equally RpfG may interact with further sensors to exert its regulatory function. Our observations suggest that alternative sensors that interact with RpfG do not recognize DSF however, as all of the genes whose transcript levels are commonly influenced by RpfF and RpfG are also under the influence of RpfC. This contrasts with work in *Xylella*, where the existence of a second DSF sensor that interacts with RpfG has been proposed (Chatterjee *et al*., 2008).

Mutation of *rpfC* in *Xcc* (and other xanthomonads) leads to an over-production of DSF but a reduction in extracellular enzyme synthesis. Previous work has shown that this elevation of DSF levels is accompanied by only a modest change in *rpfF* transcription. The observed effects of overexpression of the REC domain of RpfC on DSF synthesis have led to the proposal that RpfC sequesters RpfF via the REC domain and that the loss by mutation of RpfC releases RpfF to be active in DSF biosynthesis (He *et al*., 2006). The pathway of synthesis of the DSF family of signal molecules has been established in *Burkholderia cenocepacia* (Bi *et al*., 2012). Here the RpfF homologue BCAM0581, a bifunctional enzyme with dehydratase and thioesterase activities, catalyses the synthesis of BDSF from the acyl carrier protein (ACP) thioester of 3-hydroxydodecanoic acid. These *in vitro* findings suggest that the synthesis of DSF family signals occurs as a branch from the classic fatty acid biosynthesis pathway. Under our experimental conditions, mutation of *rpfC* also leads to elevated levels of DSF, but this is associated with a substantial increase in transcript level for *rpfF* (sixfold) as well as in genes encoding enzymes involved in fatty acid biosynthesis. This suggests that *under these conditions*, DSF synthesis is regulated primarily at the level of transcription of genes in the biosynthetic pathway (see Fig. S1). This does not exclude the possibility that RpfF sequestration by RpfC occurs at other growth phases or conditions. All the available evidence, to include work described here, indicates that regulation of DSF synthesis is largely independent of RpfG however. This suggests that transcriptional regulators whose nature remains obscure mediate the effect of RpfC on downregulation of *rpfF* gene expression. Mutation of *rpfF, rpfC* and *rpfG* genes has broader effects on gene expression than mutation of *rpfH,* which influenced expression of only four genes by a factor of fourfold. Given that RpfH has no established role in virulence, the role of this protein remains enigmatic.

### Novel genes and virulence determinants in *Xcc*

Overall the findings indicate that the Rpf system has a broad influence on transcript levels in *Xcc* (Fig. 5). Under the conditions tested, mutation of *rpfF, rpfC* or *rpfG* collectively affects the transcript levels of over 480 genes by a factor of at least fourfold.

We have demonstrated that a number of the previously annotated genes under control of the Rpf proteins are required for the full virulence of *Xcc* to Chinese radish (Fig. 5; Table S7). Many of the encoded proteins have homologues in other xanthomonads, including other *Xanthomonas* species and *Stenotrophomonas,* whereas others are more broadly conserved. It remains to be determined if the proteins have any role in the virulence of these other bacteria, some of which are human pathogens.

In addition we identified 181 genes not previously annotated within the *Xcc* 8004 genome. In total, 68 of these were regulated by the Rpf system and a subset of 16 contributed to *Xcc* virulence. Some of these genes have been identified in other bacterial genome annotations and are conserved (Table 1). We also demonstrate that three ncRNAs under the control of the Rpf/DSF system were collectively required for virulence, although loss of individual ncRNAs had no discernible effect. Such redundancy of regulatory action of small RNAs has been observed in other bacterial systems. For example, in *Vibrio cholerae* four small RNAs together with the small RNA chaperone Hfq act to control quorum sensing (Lenz *et al*., 2004).

In conclusion, our findings provide substantial new insights into the transcriptional landscape of *Xcc* and into the molecular basis of phytopathogenesis and its regulation in this model plant pathogen.

## Experimental procedures

### Bacterial strains and growth conditions

The wild-type *Xcc* 8004 and other strains have been described previously (Slater *et al*., 2000; Ryan *et al*., 2006; 2007; 2010). In-frame deletion of selected genes was carried out using pK18*mobsac* as described previously for *rpfG* (Slater *et al*., 2000). All plasmids and strains used during this study are described in Table S10. For most experiments, *Xcc* strains were grown in NYGB medium, which comprises 5 g l^−1^ bacteriological peptone (Oxoid), 3 g l^−1^ yeast extract (Difco) and 20 g l^−1^ glycerol. The antibiotics used were kanamycin (Km), rifampicin (Rif), gentamicin (Gm), spectinomycin (Sp) and tetracycline (Tc); the concentrations used are indicated.

### RNA extraction and preparation

Three independent cultures of each selected *Xanthomonas* strain were subcultured and grown to logarithmic phase (0.7–0.8 OD_600_) at 30°C in NYGB broth without selection. 800 μl of RNA protect (Qiagen) was added to 400 μl of culture and incubated at room temperature for 5 min. Cell suspensions were centrifuged, the supernatant was discarded, and pellets were stored at −80°C. After thawing, 100 μl of TE-lysozyme (400 μg ml^−1^) was added and samples were incubated at room temperature. Total RNA was isolated using the RNeasy Mini Kit (Qiagen) whereby cells were homogenized utilizing a 20-gauge needle and syringe. Samples were treated with DNase (Ambion) according to manufacturer's instructions and the removal of DNA contamination was confirmed by PCR.

### Library construction and cDNA sequencing

RNA quality was assessed on a Bioanalyser PicoChip (Agilent) and RNA quantity was measured using the RNA assay on QuBit fluorometer (Life Technologies). Ribosomal RNA was depleted with Ribo-Zero™ rRNA Removal Kits for Gram-Negative Bacteria (Epicentre). The percentage of rRNA contamination was checked on a Bioanalyser PicoChip (Agilent).

The rRNA-depleted sample was processed using the Illumina TruSeq RNA v2 sample preparation kit. In brief, the sample was chemically fragmented to ∼ 200 nt in length and the cleaved RNA fragments were primed with random hexamers into first-strand cDNA using reverse transcriptase and random primers. The RNA template was removed and a replacement strand was synthesized to generate ds cDNA. The ds cDNA was end repaired to remove the overhangs from the fragmentation into blunt ends. A single ‘A’ nucleotide was added to the 3′ ends on the blunt fragments, which is complementary to a ‘T’ nucleotide on the 3′ end of the Illumina adapters. At this stage, adapters containing 6 nt barcodes were used for different samples to allow the pooling of multiple samples together. The resulted barcoded samples were enriched by 10 cycles of PCR to amplify the amount of DNA in the library. The final cDNA libraries were sequenced on an Illumina HiSeq2000 as per manufacturer's instructions. The RNA-Seq raw data files are accessible through XanthomonasGbrowse: http://browser.tgac.bbsrc.ac.uk/cgi-bin/gb2/gbrowse/Xanthomonas_8004/.

### Computational analysis

Cluster generation was performed using the Illumina cBot and the cDNA fragments were sequenced on the Illumina HiSeq2000 following a standard protocol. The fluorescent images were processed to sequences using the Pipeline Analysis software 1.8 (Illumina). Raw sequence data obtained in Illumina FASTQ-format were first separated by their barcode sequence by comparing the first six bases with the expected barcode sequences. Successfully detected barcodes were removed from the sequence leaving reads of 30 nt in length, while reads containing no recognizable barcode sequence were discarded.

### Read mapping, annotation and quantification of transcript levels

Reads for each sample were aligned to the *Xcc* 8004 NC_007086 assembly [Integrated Microbial Genomes (IMG) database, taxon object ID 637000343] using Bowtie version 0.12.7 with default parameters. Transcript abundance was determined for the gene models annotated in the IMG *Xcc* 8004 genome release IMG/W 2.0 using the Bowtie RNA-Seq BAM alignments for each of the samples. To estimate the level of transcription for each gene, the number of reads that mapped within each annotated coding sequence (CDS) was determined. Related servers RAST (Aziz *et al*., 2008) and Prodigal (Hyatt *et al*., 2010) were also used in this way.

### Analysis of differential expression

Differential expression was assessed using Cufflinks (Trapnell *et al*., 2010; 2012). Cufflinks reports an expression value for each transcript and gene in Fragments Per Kilobase of exon model per Million mapped fragments (FPKM). A test statistic is also calculated which, after Benjamini-Hochberg correction for multiple testing was used to determine significant changes in expression between each pair of samples (false discovery rate 0.05).

### Identification of non-coding RNAs

New putative ncRNAs (i.e. ncRNAs not previously reported or previously identified by Rfam) were manually identified using the genome browser Artemis (Rutherford *et al*., 2000) and an *in silico* approach used by Mandin *et al*. (2007). Specifically, intergenic regions not matching annotated genes, but showing contiguous coverage by RNA-Seq reads (i.e. regions that contain at least 100 bp completely covered by RNA-Seq reads) were designated putative ncRNAs. Further, RNA-Seq reads that did not cover an entire annotated CDS, but showed partial contiguous coverage within a CDS, were also designated as putative ncRNAs. All ncRNAs, identified by RNA-Seq, but with no matches to the Rfam database were designated ‘putative ncRNA’ and received designations from sRNAXcc-5 to sRNAXcc-29 following the nomenclature described by Chen *et al*. (2011).

### Rfam scan

The *Rfam* database version 10.0 was downloaded from http://ftp://ftp.sanger.ac.uk/pub/databases/Rfam/10.0/. To scan the *Xcc* genome for known non-coding RNAs the *Rfam* provided Perl script rfam_scan.pl with an e-value cut-off of 100 was used.

### Quantitative real-time PCR

Quantitative RT-PCRs were used to validate RNA-Seq data. Reverse transcription PCR was achieved using a cDNA synthesis kit (Promega) according to the manufacturer's instructions. Specific RT-PCR primers were used to amplify central fragments of approximately 200 bp in length from different genes. Semi-quantitative RT-PCRs were completed using 250 ng μl^−1^ cDNA template and PCR Mastermix (Promega) for 24–36 cycles. For qRT-PCRs, quantification of gene expression and melting curve analysis were completed using a LightCycler (Roche) and Platinum SYBR Green qPCR Supermix-UDG (Invitrogen) was used according to manufacturer's instructions. The constitutively expressed housekeeping gene, 16S rRNA was used as a reference to standardize all samples and replicates.

### Construction of strains with mutations in genes regulated by the Rpf/DSF signalling system

Mutants with single Tn*5gusA* insertions in genes of interest were identified within a library of such mutants generated following the procedure of Sharma and Signer (1990). First the cosmid vector pLAFR1::Tn*5gus*A5 was introduced into *Xcc* 8004 by triparental mating with pRK2073 as helper. Plasmid pPH1JI (which is incompatible with pLAFR1) was then introduced by mating from *Xcc* 8005/pPH1JI. Strains with a transposon insertion in the chromosome were selected by plating on 25 μg ml^−1^ Km, 50 μg ml^−1^, Rif, 5 μg ml^−1^ Gm and 50 μg ml^−1^ Sp. The colonies were then checked for Tc-sensitivity by streaking on plates containing 5 μg ml^−1^ Tc. Individual colonies were picked from a large number of such mating and selection procedures. The genomic positions of the transposon insertion within this library of mutants were established by TAIL-PCR and sequencing for comparison with the full genome sequence of *Xcc* strain 8004 (Qian *et al*., 2005). After lysing cells at 95°C, sequences flanking the site of transposon insertion were amplified with a transposon-specific primer and an arbitrary primer, followed by a second amplification using a nested transposon-specific primer and a primer corresponding to a non-random portion of the arbitrary primer used in the first PCR. A third nested transposon-specific primer was used for sequencing reactions.

Mutants were also created by the disruption of genes with the use of the plasmid pK18*mob* as described previously for *Xcc* (Dow *et al*., 2003). An internal fragment of approximately 500 bp of the gene to be disrupted was amplified by PCR using *Xcc* chromosomal DNA as template, and cloned by using the TOPO TA cloning kit (Invitrogen). The identity of the cloned fragment was confirmed by sequencing. The fragment was excised from this construct with EcoRI, which cuts at sites flanking the insert. This EcoRI fragment was ligated into pK18*mobkan*. This construct was conjugated from *Escherichia coli* DH5α into *Xcc* by triparental mating by using the helper plasmid pRK2073. Mutants were selected on plates containing Rif (50 μg ml^−1^) and Km (12.5 μg ml^−1^). Mutants were screened for disruption of the selected gene by PCR (Table S10). This method was used to create mutants in the *rpfG* background. For other double mutations, a derivative of pK18*mobkan* was used in which the Km resistance-conferring gene was excised and replaced with a Tc-resistance determinant from pLAFR3 to create pK18*mobtet*.

### DNA manipulation

Molecular biological methods such as isolation of plasmid and chromosomal DNA, PCR, plasmid transformation as well as restriction digestion were carried out using standard protocols (Sambrook *et al*., 1989). PCR products were cleaned using the Qiaquick PCR purification kit (Qiagen) and DNA fragments were recovered from agarose gels using Qiaquick minielute gel purification kit (Qiagen). Oligonucleotide primers were purchased from Sigma-Genosys.

### Virulence assays

The virulence of *Xcc* to Chinese radish was estimated after bacteria were introduced into the leaves by leaf clipping as previously detailed (Dow *et al*., 2003; Ryan *et al*., 2007). Bacteria grown overnight in NYGB medium were washed and resuspended in water to an OD at 600 nm of 0.001. For leaf clipping the last completely expanded leaf was cut with scissors dipped in the bacterial suspensions. Thirty leaves were inoculated for each strain tested. Lesion length was measured 14 days after inoculation. Each strain was tested in at least four separate experiments.
