# Supplementary material for: High-resolution transcriptional analysis of the regulatory influence of cell-to-cell signalling reveals novel genes that contribute to Xanthomonas phytopathogenesis
Source: Mol Microbiol. 2013 Apr 26;88(6):1058–69. doi: 10.1111/mmi.12229 (PMC3744752; doi:10.1111/mmi.12229)
Supplement: Supplementary file 1 [file mmi0088-1058-SD1.pdf]

SUPPORTING FIGURE AND TABLES

Fig. S1. Mutation of *rpfC* leads to elevated levels of DSF, but this is associated with a substantial increase in transcript level for *rpfF.* Transcript level of the gene that encodes the RpfF protein was measured in wild-type strain (8004), *rpfC* and *rpfG* mutant backgrounds by qRT-PCR as described in the Experimental procedures. Bars show mean fold changes obtained from three independent experiments.

Table S1. Summary of sequencing data for the *Xcc* cDNA samples.

Table S2. List of novel transcripts discovered by RNA-Seq in *Xanthomonas campestris* pv. *campestris* 8004 genome. Novel transcripts include those found in intergenic regions and annotated genes found in other Xanthomonads.

Table S3. Summary of intergenic non-coding RNA candidates in *Xcc.*

Table S4. List of genes differentially expressed in *rpfF*, *rpfC*, *rpfG* and/or *rpfH* mutant backgrounds compared to wild-type (fold change ≥ 4). Significantly differentially expressed genes were determined by Cufflinks after Benjamini-Hochberg correction. The fold change is the ratio of mutant FPKM to wild-type FPKM. The genes were ranked by size of their fold change.

Table S5. Genes divergently regulated by different Rpf proteins. Significantly differentially expressed genes were determined by Cufflinks, after Benjamini-Hochberg correction. The fold change is the ratio of mutant FPKM to wild-type FPKM. The genes were ranked on their fold change.

Table S6. Quantitative RT-PCR validation of data generated by RNA-Seq. The Table shows fold changes in *rpfF*, *rpfC* and *rpfG* mutants compared to wild-type for a selection of genes as measured by qRT-PCR and RNA-Seq.

Table S7. The effects of mutation of previously annotated genes in *Xcc* identified to be regulated by RpfF, RpfC and/or RpfG on virulence. The virulence of each mutant was tested by measurement of the lesion length after bacteria were introduced into the vascular system of Chinese radish by leaf clipping.

Table S8. Effects of combinatorial mutation of previously annotated genes on the virulence of *Xcc* to Chinese radish. The virulence of each mutant was tested by measurement of the lesion length after bacteria were introduced into the vascular system of Chinese radish by leaf clipping. Double mutations did not give

rise to an additive effect on virulence.

Table S9. List of the 68 unannotated genes regulated by RpfF, RpfC and/or RpfG and the influence of mutation of these genes on virulence of *Xcc* to Chinese radish.

Table S10. Table of strains and plasmids used in this study.

Fig. S1. Mutation of *rpfC* leads to elevated levels of DSF, but this is associated with a substantial increase in transcript level for *rpfF.* Transcript level of the gene that encodes the RpfF protein was measured in wild-type strain (8004), *rpfC* and *rpfG* mutant backgrounds by qRT-PCR as described in the Experimental procedures. Bars show mean fold changes obtained from three independent experiments.

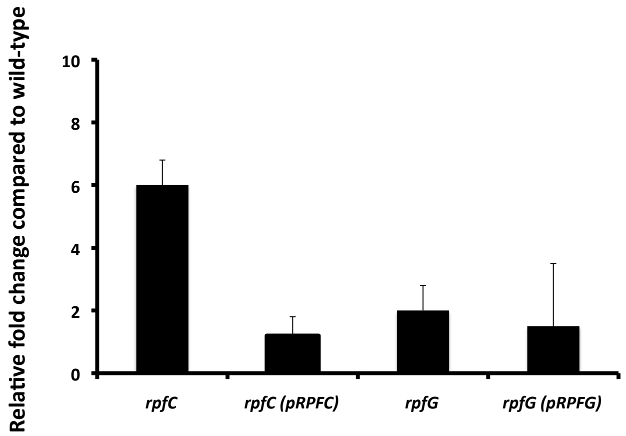

Table S1. Summary of sequencing data for the *Xcc* cDNA samples.

Sequenced sample*	Average read size, bp	No. of total mapped reads	No. of total mapped bps (× 106)	No. of genes mapped	rRNA reads (% of all mapped reads)	otherRNA reads (% of all mapped reads)
Wild-type (8004) [1]	41	23,384,369	22,935,457	4,314	0.34	6.88
Wild-type (8004) [2]	55	139,959,239	70,091,568	3,963	0.26	1.45
Wild-type (8004) [3]	41	69,979,620	35,045,784	4,318	0.19	0.06
rpfF [1]	44	38,499,783	37,989,891	4,319	0.43	13.2
rpfF [2]	39	53,182,549	49,316,819	4,318	0.83	10.1
rpfF [3]	49	53,943,623	53,240,572	4,319	0.08	0.03
rpfC [1]	39	30,302,875	29,719,078	4,317	0.11	7.28
rpfC [2]	39	46,653,080	23,363,856	4,318	0.12	0.89
rpfC [3]	41	49,907,111	45,501,222	4,316	0.09	0.67
rpfG [1]	51	42,610,153	41,694,010	4,319	1.18	5.02
rpfG [2]	47	55,983,696	28,036,627	4,319	0.51	2.81
rpfG [3]	48	51,897,564	50,987,056	4,318	0.21	2.01
rpfH [1]	51	29,845,577	29,187,183	4,315	0.25	1.34
rpfH [2]	64	32,976,059	32,351,133	4,313	0.24	0.85
rpfH [3]	55	26,608,823	25,566,428	4,250	2.32	3.65
**Average total**	**47**	**49,715,608**	**38,335,112**	**4,289**	**0.48**	**3.75**

*Indicates the Xanthomonas campestris strain and biological replicate number [1 or 2 or 3].

Table S2. List of novel transcripts discovered by RNA-Seq in *Xanthomonas campestris* pv. *campestris* 8004 genome. Novel transcripts include those found in intergenic regions and annotated genes found in other Xanthomonads.

Designation[1]	Start[2]	End[2]	Length (nt)	Predicted function[3]	Region[4]
TID1	49789	50013	225	hypothetical protein	IN
TID2	50022	50402	381	hypothetical protein	IN
TID3	77017	77220	204	hypothetical protein	IN
TID4	78431	79402	972	regulatory protein	OT
TID5	80767	81213	447	hypothetical protein	IN
TID6	115813	116373	561	chloroacetaldehyde dehydrogenase	IN
TID7	137593	137976	384	hypothetical protein	IN
TID8	163742	164440	699	hypothetical protein	IN
TID9	164621	164956	336	hypothetical protein	IN
TID10	171586	172836	1251	hypothetical protein	IN
TID11	184341	184565	225	hypothetical protein	IN
TID12	248876	249256	381	hypothetical protein	OT
TID13	329026	329337	312	hypothetical protein	IN
TID14	330018	330632	615	hypothetical protein	OT
TID15	343040	343153	114	oxidoreductase	IN
TID16	394150	394791	642	hypothetical protein	OT
TID17	404902	405264	363	hypothetical protein	IN
TID18	407169	407657	489	hypothetical protein	IN
TID19	407739	408065	327	hypothetical protein	IN
TID20	420032	420388	357	hypothetical protein	IN
TID21	466526	467683	1158	hypothetical protein	IN
TID22	522841	523053	213	hypothetical protein	IN
TID23	523180	523356	177	pseudogene	PP
TID24	549720	549977	258	hypothetical protein	IN

TID25	549882	550175	294	hypothetical protein	IN
TID26	550132	551613	1482	hypothetical protein	IN
TID27	553163	553849	687	hypothetical protein	OT
TID28	577673	577957	285	quaternary ammonium compound-resistance protein, SugE	OT
TID29	601193	601891	699	hypothetical protein	IN
TID30	686074	686502	429	hypothetical protein	OT
TID31	689924	690088	165	hypothetical protein	OT
TID32	710291	710434	144	hypothetical protein	OT
TID33	716197	716328	132	hypothetical protein	IN
TID34	745782	746678	897	hypothetical protein	IN
TID35	825385	825765	381	hypothetical protein	OT
TID36	838746	839714	969	hypothetical protein	IN
TID37	843819	844055	237	hypothetical protein	OT
TID38	913310	913897	588	hypothetical protein	IN
TID39	936331	936711	381	hypothetical protein	OT
TID40	942479	942856	378	hypothetical protein	IN
TID41	942977	943399	423	hypothetical protein	PP
TID42	943400	944080	681	hypothetical protein	IN
TID43	968889	969158	270	hypothetical protein	OT
TID44	1006360	1007040	681	hypothetical protein	IN
TID45	1040519	1041289	771	hypothetical protein	IN
TID46	1059945	1060547	603	hypothetical protein	OT
TID47	1089233	1089613	381	hypothetical protein	OT
TID48	1091488	1091868	381	hypothetical protein	OT
TID49	1161375	1161761	387	hypothetical protein	OT
TID50	1182170	1182316	147	hypothetical protein	IN
TID51	1233244	1233663	420	hypothetical protein	IN
TID52	1241824	1242060	237	hypothetical protein	IN

TID53	1246507	1246767	261	hypothetical protein	OT
TID54	1246828	1247094	267	hypothetical protein	IN
TID55	1247127	1247693	567	hypothetical protein	IN
TID56	1248791	1249162	372	hypothetical protein	IN
TID57	1251696	1251986	291	hypothetical protein	IN
TID58	1254044	1254403	360	hypothetical protein	IN
TID59	1258740	1258964	225	hypothetical protein	IN
TID60	1265318	1265524	207	hypothetical protein	OT
TID61	1388066	1388383	318	hypothetical protein	OT
TID62	1446771	1446920	150	hypothetical protein	OT
TID63	1468983	1469114	132	hypothetical protein	IN
TID64	1568785	1569600	816	hypothetical protein	OT
TID65	1576448	1576576	129	hypothetical protein	IN
TID66	1578109	1578369	261	hypothetical protein	OT
TID67	1740863	1741468	606	hypothetical protein	OT
			348	hypothetical protein	OT
TID68	1744147	1744494		hypothetical protein	OT
TID69	1791905	1792030	126	hypothetical protein	OT
TID70	1832716	1832988	273	hypothetical protein	IN
TID71	1834469	1834759	291	hypothetical protein	IN
TID72	1876483	1877004	522	xcc-b100_1603	IN
TID73	1888167	1888415	249	hypothetical protein	IN
TID74	2084040	2084192	153	hypothetical protein	OT
TID75	2193981	2194241	261	hypothetical protein	OT
TID76	2297488	2297586	99	hypothetical protein	OT
TID77	2330603	2330695	93	hypothetical protein	OT
TID78	2335517	2335876	360	hypothetical protein	OT
TID79	2374830	2375054	225	hypothetical protein	OT
TID80	2416778	2416984	207	hypothetical protein	IN

TID81	2422070	2422750	681	hypothetical protein	PP
TID82	2452520	2452753	234	hypothetical protein	OT
TID83	2467081	2467329	249	hypothetical protein	IN
TID84	2474282	2474494	213	hypothetical protein	OT
TID85	2484895	2485155	261	hypothetical protein	OT
TID86	2486779	2487129	351	hypothetical protein	IN
TID87	2489339	2490940	1602	hypothetical protein	IN
TID88	2491280	2492113	834	hypothetical protein	IN
TID89	2531915	2532082	168	hypothetical protein	OT
TID90	2534397	2534768	372	hypothetical protein	OT
TID91	2536676	2536984	309	hypothetical protein	IN
TID92	2537956	2538264	309	hypothetical protein	IN
TID93	2540172	2540459	288	hypothetical protein	OT
TID94	2542891	2543073	183	hypothetical protein	OT
TID95	2556319	2556555	237	hypothetical protein	OT
TID96	2559681	2560061	381	hypothetical protein	OT
TID97	2566469	2566744	276	hypothetical protein	IN
TID98	2573230	2573448	219	hypothetical protein	IN
TID99	2584403	2584846	444	hypothetical protein	OT
TID100	2611087	2611344	258	hypothetical protein	OT
TID101	2764514	2764882	369	hypothetical protein	OT
TID102	2768924	2769202	279	hypothetical protein	IN
TID103	2796093	2796428	336	hypothetical protein	IN
TID104	2886304	2886393	90	hypothetical protein	IN
TID105	2896689	2898389	1701	outer membrane hemolysin activator protein	IN
TID106	2927534	2927761	228	hypothetical protein	OT
TID107	2947726	2947938	213	hypothetical protein	OT
TID108	3007215	3007325	111	hypothetical protein	OT

TID109	3017100	3017309	210	hypothetical protein	IN
TID110	3095886	3096053	168	hypothetical protein	OT
TID111	3115125	3115253	129	hypothetical protein	OT
TID112	3117086	3117403	318	hypothetical protein	OT
TID113	3122764	3122985	222	hypothetical protein	IN
TID114	3130655	3130939	285	hypothetical protein	IN
TID115	3130909	3131289	381	hypothetical protein	IN
TID116	3131695	3132516	822	hypothetical protein	
TID117	3132546	3132713	168	hypothetical protein	IN
TID118	3140515	3140955	441	hypothetical protein	OT
TID119	3142126	3142941	816	hypothetical protein	IN
TID120	3148912	3149193	282	hypothetical protein	IN
TID121	3159345	3160106	762	hypothetical protein	IN
TID122	3163929	3164765	837	hypothetical protein	OT
TID123	3170824	3171354	531	hypothetical protein	OT
TID124	3287554	3287802	249	hypothetical protein	IN
TID125	3344597	3344863	267	hypothetical protein	OT
TID126	3347349	3347600	252	hypothetical protein	IN
TID127	3355411	3355674	264	hypothetical protein	OT
TID128	3369419	3369646	228	hypothetical protein	OT
TID129	3387441	3387536	96	hypothetical protein	IN
TID130	3519895	3520152	258	hypothetical protein	IN
TID131	3580613	3580726	114	hypothetical protein	OT
TID132	3592803	3593480	678	hypothetical protein	OT
TID133	3596048	3596203	156	hypothetical protein	OT
TID134	3620156	3620248	93	hypothetical protein	IN
TID135	3627629	3627952	324	hypothetical protein	OT
TID136	3630004	3630723	720	hypothetical protein	OT
TID137	3669579	3669764	186	hypothetical protein	IN

TID138	3669779	3670048	270	hypothetical protein	IN
TID139	3669996	3670376	381	hypothetical protein	IN
TID140	3677814	3677945	132	hypothetical protein	IN
TID141	3749515	3749853	339	hypothetical protein	IN
TID142	3762527	3762619	93	hypothetical protein	IN
TID143	3773215	3773388	174	hypothetical protein	OT
TID144	3878843	3879049	207	hypothetical protein	OT
TID145	3934518	3935066	549	hypothetical protein	IN
TID146	3959221	3959445	225	hypothetical protein	IN
TID147	4015571	4016146	576	hypothetical protein	IN
TID148	4063928	4064359	432	hypothetical protein	OT
TID149	4142182	4142433	252	hypothetical protein	IN
TID150	4173581	4173727	147	hypothetical protein	OT
TID151	4214753	4215184	432	hypothetical protein	OT
TID152	4265199	4265864	666	hypothetical protein	IN
TID153	4305065	4305484	420	hypothetical protein	IN
TID154	4363454	4363543	90	hypothetical protein	IN
TID155	4375383	4376636	1254	hypothetical protein	OT
TID156	4413491	4414270	780	hypothetical protein	OT
TID157	4553290	4553514	225	hypothetical protein	IN
TID158	4568882	4569238	357	hypothetical protein	IN
TID159	4621944	4622825	882	hypothetical protein	IN
TID160	4624524	4625456	933	hypothetical protein	IN
TID161	4660170	4660412	243	hypothetical protein	OT
TID162	4741178	4741453	276	hypothetical protein	IN
TID163	4752624	4752758	135	hypothetical protein	IN
TID164	4819804	4820448	645	hypothetical protein	OT
TID165	4846309	4846626	318	hypothetical protein	IN
TID166	4862456	4863091	636	hypothetical protein	OT

TID167	4915761	4915937	177	hypothetical protein	IN
TID168	4933037	4933282	246	hypothetical protein	OT
TID169	4946550	4946888	339	hypothetical protein	OT
TID170	4963852	4964841	990	hypothetical protein	OT
TID171	4967753	4967986	234	hypothetical protein	IN
TID172	4991518	4991631	114	hypothetical protein	OT
TID173	5043221	5043934	714	hypothetical protein	IN
TID174	5065515	5065613	99	hypothetical protein	IN
TID175	5065734	5066060	327	pseudogene	PP
TID176	5066234	5066443	210	hypothetical protein	IN
TID177	5068515	5068715	201	hypothetical protein	OT
TID178	5071301	5072137	837	hypothetical protein	IN
TID179	5072267	5072725	459	hypothetical protein	IN
TID180	5074874	5075731	858	Sir2-like protein	IN
TID181	5134322	5134450	129	hypothetical protein	OT

[1]Designation of novel transcripts

[2]Annotation taken from Qian et al., [22]

[3]Best BLAST hit after searching sequenced bacterial genomes.

[4]Genomic contex of transcriped regions - Intergenic region (IN), Overlapping with annotated gene (OT), pseudogenes (PP).

Table S3. Summary of intergenic non-coding RNA candidates in *Xcc.*

Designation[a]	Start[b]	End[b]	Intergenic region start[b]	Intergenic region end[b]	Length (nt)	5' flanking gene[b]
sRNAXcc-5	46614	46672	46187	46765	59	XC_0035
sRNAXcc-6	79055	79114	78434	79468	60	XC_0063
sRNAXcc-7	81165	81218	80668	81391	54	XC_0065
sRNAXcc-8	330632	330722	330561	331061	90	XC_0277
sRNAXcc-9	337655	337739	337726	338123	85	XC_0284
sRNAXcc-10	551604	551667	551613	551723	64	XC_0463
sRNAXcc-11	1233305	1233394	1233059	1233696	90	XC_1018
sRNAXcc-12	1265456	1265545	1264862	1265512	90	XC_1048
sRNAXcc-13	1578152	1578285	1577933	1578188	134	XC_1289
sRNAXcc-14	1791899	1791966	1791804	1792027	68	XC_1485
sRNAXcc-15	2566429	2566502	2566337	2566943	74	XC_2138
sRNAXcc-16	2566599	2566688	2566337	2566943	90	XC_2138
sRNAXcc-17	2611091	2611200	2611075	2611524	110	XC_2165
sRNAXcc-18	2746312	2746409	2745453	2746453	98	XC_2278
sRNAXcc-19	2927371	2927572	2927467	2927753	201	XC_2416
sRNAXcc-20	3148927	3149006	3148381	3149221	80	XC_2607
sRNAXcc-21	3387384	3387473	3387345	3387574	90	XC_2816
sRNAXcc-22	3519856	3519935	3519454	3520541	80	XC_2934
sRNAXcc-23	3749596	3749685	3749450	3750274	90	XC_3137
sRNAXcc-24	3959367	3959450	3958328	3959469	84	XC_3304
sRNAXcc-25	4575554	4575632	4575597	4575821	79	XC_3870
sRNAXcc-26	4858203	4858294	4858292	4858634	92	XC_4123
sRNAXcc-27	4991506	4991585	4991402	4991628	80	XC_4221
sRNAXcc-28	5068506	5068612	5068008	5068712	107	XC_4273

aDesignation adapted and continued from Chen and colleagues (2011).
bAnnotation taken from Qian et al., (2005).

Table S4. List of genes differentially expressed in *rpfF*, *rpfC*, *rpfG* and/or *rpfH* mutant backgrounds compared to wild-type (fold change ≥ 4). Significantly differentially expressed genes were determined by Cufflinks after Benjamini-Hochberg correction. The fold change is the ratio of mutant FPKM to wild-type FPKM. The genes were ranked by size of their fold change.

Gene locus[a]	Function[b]	Change Fold (log2)[c]			
		RpfC	RpfF	RpfG	RpfH
XC_0025	hypothetical protein	-2.56			
XC_0026	cellulase			-2.76	
XC_0027	cellulase			-2.67	
XC_0060	NAD(P)H oxidoreductase	-8.59	-8.90		
XC_0061	hypothetical protein		INF		
XC_0062	hypothetical protein	-7.73	-7.75		
XC_0063	regulatory protein cII	-10.72	-10.71		
XC_0064	hypothetical protein	-5.32	-6.26		
XC_0065	hypothetical protein	-10.81	-10.64		
XC_0082	AtsE protein	-2.76			
XC_0083	thioredoxin	-2.88			
XC_0084	proline/betaine transporter	-2.44			
XC_0093	hypothetical protein	2.54			
XC_0094	TldD protein	2.99			
XC_0107	hypothetical protein	-2.40			
XC_0108	hypothetical protein	-2.75			
XC_0109	ATP-dependent DNA ligase	-2.88			
XC_0112	hypothetical protein		INF		

XC_0113	two-component system sensor protein	-10.57	-9.49	
XC_0114	two-component system regulatory protein		-10.06	
XC_0117	hypothetical protein			-2.34
XC_0128	hypothetical protein	-11.17		
XC_0129	hypothetical protein	-8.85	-9.39	
XC_0130	hypothetical protein		INF	
XC_0131	hypothetical protein	-8.92	-9.44	
XC_0132	deoxycytidylate deaminase	-9.18	-9.36	
XC_0136	RhsD protein	-11.20	-11.19	
XC_0223	hypothetical protein	-10.19	-9.86	
XC_0224	methyltransferase	-9.96	-10.00	
XC_0225	hypothetical protein	-10.53	-10.27	
XC_0230	hypothetical protein	-3.52	-3.62	
XC_0286	chemotaxis protein	2.95		
XC_0296	oxidoreductase	-5.94		
XC_0323	transcriptional regulator		INF	
XC_0324	insect-type dehydrogenase		INF	
XC_0331	hypothetical protein		INF	
XC_0343	hypothetical protein	-10.02	-10.20	
XC_0344	hypothetical protein	-3.81	-3.29	
XC_0347	hypothetical protein		INF	
XC_0350	hypothetical protein	-10.84	-6.81	
XC_0351	hypothetical protein	-10.30	-10.20	
XC_0412	IS1404 transposase protein A	3.30		
XC_0475	hypothetical protein	-9.51	-8.72	
XC_0476	type I restriction-modification system endonuclease	-8.92	-8.87	
XC_0477	restriction modification system specificity subunit	-9.32	-9.44	

XC_0478	anticodon nuclease	-9.41	-9.54	
XC_0479	DNA-binding protein	-11.12	-11.38	
XC_0480	type I site-specific deoxyribonuclease	-10.04	-10.05	
XC_0543	hypothetical protein			-2.63
XC_0544	hypothetical protein	3.99	3.66	
XC_0547	hypothetical protein	-2.76		
XC_0549	hypothetical protein	-3.47		
XC_0585	hypothetical protein	-4.23	-4.29	
XC_0586	hypothetical protein	-3.24	-3.29	
XC_0593	hypothetical protein	-2.33		
XC_0598	hypothetical protein	-2.84		
XC_0599	hypothetical protein	-2.80		
XC_0637	histidine kinase/response regulator hybrid protein	3.11		
XC_0638	chemotaxis protein			-3.24
XC_0639	cellulase			-6.92
XC_0710	hypothetical protein	-9.64	-9.73	
XC_0723	hypothetical protein	-2.41		
XC_0727	hypothetical protein	-2.62		
XC_0783	cellulase S			-4.17
XC_0811	porin	-2.43		
XC_0817	hypothetical protein		INF	
XC_0818	sensor histidine kinase	2.44		
XC_0849	TonB-dependent receptor	-8.40	-7.70	
XC_0856	hypothetical protein	-10.53	-10.69	
XC_0857	hypothetical protein	-9.43	-9.49	
XC_0861	hypothetical protein		INF	
XC_0863	hypothetical protein	-8.56	-8.62	

XC_0864	VirB6 protein	-10.54	-10.41	
XC_0865	hypothetical protein	-8.48	-8.61	
XC_0866	hypothetical protein	-10.05	-10.22	
XC_0867	hypothetical protein	-10.20	-10.35	
XC_0868	VirB6 protein	-9.84	-9.83	
XC_0870	hypothetical protein	-10.96	-10.19	
XC_0871	hypothetical protein		-10.89	
XC_0914	hypothetical protein		-5.73	
XC_0915	TonB-like protein	-9.61	-9.39	
XC_0916	BlaI family transcriptional regulator	-10.92	-10.51	
XC_0922	reductase	-10.87	-10.82	
XC_0923	transcriptional regulator		INF	
XC_0924	outer membrane receptor for ferric iron uptake		INF	
XC_0925	outer membrane receptor for ferric iron uptake		INF	
XC_0967	hypothetical protein		INF	
XC_0980	hypothetical protein	-2.28		
XC_1004	TonB-dependent receptor	2.53		-3.13
XC_1018	phage-related integrase	-11.26	-11.32	
XC_1019	hypothetical protein	-12.68	-12.83	-2.46
XC_1021	hypothetical protein	-5.88	-5.38	
XC_1022	regulatory protein bphR	-3.01		
XC_1023	hypothetical protein	-10.20	-10.33	
XC_1027	VirB6 protein	-9.19	-9.19	
XC_1028	hypothetical protein	-9.71	-8.96	
XC_1029	hypothetical protein		INF	
XC_1030	hypothetical protein	-6.20	-6.14	
XC_1036	hypothetical protein		INF	

XC_1037	hypothetical protein	-10.35	-10.55	
XC_1038	hypothetical protein		INF	
XC_1039	hypothetical protein	-10.92	-11.06	
XC_1040	hypothetical protein	-7.46	-7.81	
XC_1041	hypothetical protein	-9.52	-9.59	
XC_1042	hypothetical protein		INF	
XC_1057	fimbrial assembly protein	-8.73	-8.15	
XC_1058	pilin	-11.12	-11.28	
XC_1059	pilin	-9.14	-8.84	
XC_1088	ATP-dependent Clp protease subunit	2.52		
XC_1154	membrane transport protein	-2.48		
XC_1166	glucokinase	-2.31		
XC_1185	pilus biogenesis protein	2.63		
XC_1190	hypothetical protein	2.77		
XC_1194	hypothetical protein	-2.54		
XC_1201	RebB protein	3.62	3.78	
XC_1202	hypothetical protein	2.71		
XC_1213	virulence protein	-4.02	-4.11	
XC_1264	hypothetical protein	-3.54		
XC_1291	endoproteinase ArgC			-3.49
XC_1292	endoproteinase ArgC			-4.54
XC_1298	pectate lyase II			-2.05
XC_1300	quinol oxidase subunit I	3.16		2.94
XC_1301	quinol oxidase subunit II	2.81		2.17
XC_1379	hypothetical protein	-9.41	-9.59	
XC_1380	McrB-like protein	-9.60	-9.61	
XC_1381	hypothetical protein	-11.31	-10.92	

XC_1391	hypothetical protein			-2.69
XC_1409	chemotaxis-specific methylesterase	2.34		
XC_1410	response regulator for chemotaxis	3.07		
XC_1411	response regulator	3.17		
XC_1412	chemotaxis protein	2.56		
XC_1413	chemotaxis protein	3.15		
XC_1414	chemotaxis histidine protein kinase	2.93		
XC_1415	hypothetical protein	2.99		
XC_1422	cysteine protease		INF	
XC_1423	hypothetical protein	3.47		
XC_1441	hypothetical protein		INF	
XC_1442	serine protease		INF	
XC_1451	TonB-dependent receptor	-2.26		-2.95
XC_1471	hypothetical protein	-3.96		
XC_1515	extracellular protease			-4.20
XC_1549	hypothetical protein	-8.09	-8.58	
XC_1621	pre-pilin like leader sequence	-9.97	-9.77	
XC_1622	pre-pilin leader sequence	-9.78	-9.56	
XC_1623	hypothetical protein	-9.19	-9.19	
XC_1624	PilX protein	-10.72	-10.80	
XC_1625	PilY1 protein	-10.15	-10.20	
XC_1626	type IV pilin	-10.47	-10.26	
XC_1631	hypothetical protein		INF	
XC_1632	VirB8 protein	-9.64	-4.84	
XC_1633	VirB9 protein	-10.26	-10.44	
XC_1634	VirB10 protein	-10.42	-10.39	
XC_1635	VirB11 protein	-9.36	-9.39	

XC_1636	VirB1 protein	-9.39	-9.55	
XC_1637	VirB2 protein	-10.31	-10.22	
XC_1638	VirB3 protein	-10.45	-10.60	
XC_1639	VirB4 protein	-9.32	-9.30	
XC_1640	hypothetical protein	-4.25	-5.28	
XC_1660	GumD protein	-2.28		
XC_1719	hypothetical protein	-9.37	-9.46	
XC_1728	uracil phosphoribosyltransferase	-4.67		
XC_1732	hypothetical protein			4.59
XC_1766	transcriptional regulator	2.40		
XC_1790	repressor	-2.32		
XC_1853	hypothetical protein	-3.38		
XC_1880	gamma-glutamyl phosphate reductase	-6.48	-6.47	
XC_1945	transporter	-8.19	-8.39	
XC_2009	hypothetical protein	-3.52	-4.11	
XC_2011	IS1477 transposase	3.43		
XC_2013	sensor kinase	-10.49	-10.66	
XC_2014	hypothetical protein	-1.79769e+308	INF	
XC_2015	hypothetical protein	-9.42	-8.53	
XC_2016	VirB6 protein	-10.61	-9.55	
XC_2017	hypothetical protein		-11.31	
XC_2018	hypothetical protein	-6.04	-5.97	
XC_2019	hypothetical protein	-3.45	-3.50	
XC_2041	hypothetical protein	-3.07		
XC_2043	hypothetical protein	-2.94		
XC_2044	hypothetical protein	-9.22	-9.29	
XC_2045	hypothetical protein	-4.13		

XC_2046	plasmid-like protein	-2.43		
XC_2048	hypothetical protein	-2.58		
XC_2055	hypothetical protein	-9.62	-9.18	
XC_2056	hypothetical protein		INF	
XC_2058	hypothetical protein	-7.14	-8.06	
XC_2059	hypothetical protein	-10.82	-10.87	
XC_2060	hypothetical protein	-10.41	-10.44	
XC_2061	hypothetical protein		INF	
XC_2074	hypothetical protein	-8.43	-8.60	
XC_2075	transcriptional regulator	-9.90	-10.02	
XC_2078	hypothetical protein	-3.33	-3.44	
XC_2079	hypothetical protein	-3.11		
XC_2085	hypothetical protein	-11.87	-11.92	
XC_2086	DNA helicase-like protein	-10.02	-9.87	
XC_2087	tannase	-12.15	-12.14	-3.22
XC_2088	hypothetical protein	-10.31	-10.70	-4.23
XC_2089	hypothetical protein	-8.55	-9.27	
XC_2090	coproporphyrinogen III oxidase	-5.43	-5.08	
XC_2097	hypothetical protein	-2.57		
XC_2106	hypothetical protein	-10.93	-8.47	
XC_2107	replication initiation protein	-4.22		
XC_2112	adsorption protein	-3.45		
XC_2113	minor coat protein		-7.14	
XC_2116	hypothetical protein		INF	
XC_2123	hypothetical protein	-8.34	-8.52	
XC_2124	hypothetical protein		INF	
XC_2126	hypothetical protein	-2.73		

XC_2128	cyclic beta 1-2 glucan synthetase	-8.62	-4.03		
XC_2134	IS1480 transposase	2.60			
XC_2135	hypothetical protein			2.34	
XC_2156	nodulation related protein	-2.49			
XC_2159	serine protease	2.81			
XC_2193	hypothetical protein	-2.63			
XC_2223	chemotaxis protein	2.69		-2.50	
XC_2224	hypothetical protein	3.10			
XC_2230	hypothetical protein	2.34		-2.32	
XC_2231	flagellar protein	2.36		-2.77	
XC_2232	flagellar basal body P-ring biosynthesis protein FlgA			-2.43	
XC_2233	chemotaxis protein	2.24			
XC_2234	flagellar basal-body rod protein FlgB			-4.41	-2.37
XC_2235	flagellar basal body rod protein FlgC			-4.87	
XC_2236	flagellar basal body rod modification protein			-4.22	
XC_2237	flagellar hook protein FlgE			-4.34	
XC_2238	flagellar basal body rod protein FlgF			-3.09	
XC_2239	flagellar basal body rod protein FlgG			-4.49	
XC_2240	flagellar basal body L-ring protein			-4.34	
XC_2241	flagellar basal body P-ring biosynthesis protein FlgA			-3.35	
XC_2242	flagellar rod assembly protein/muramidase FlgJ			-2.96	
XC_2243	flagellar hook-associated protein FlgK			-3.28	
XC_2244	flagellar hook-associated protein FlgL			-2.86	
XC_2245	flagellin	2.49		-4.40	
XC_2246	flagellar protein			-3.54	
XC_2247	flagellar protein			-2.40	
XC_2251	RNA polymerase sigma-54 factor			-2.73	

XC_2259	flagellar protein			-2.76	
XC_2260	flagellar MS-ring protein			-2.92	
XC_2261	flagellar protein			-3.33	
XC_2262	flagellar protein			-2.70	
XC_2263	flagellar protein			-2.10	
XC_2264	flagellar FliJ protein			-2.81	
XC_2265	flagellar protein			-2.97	
XC_2266	flagellar biosynthesis protein			-3.05	
XC_2267	flagellar motor switch protein FliM			-3.08	
XC_2268	flagellar protein			-2.25	
XC_2269	flagellar protein			-2.34	
XC_2272	flagellar biosynthesis			-2.89	
XC_2277	flagellar biosynthesis protein FlhB			-2.34	
XC_2278	flagellar biosynthesis protein FlhA			-2.43	
XC_2279	flagellar biosynthesis regulator FlhF			-4.16	
XC_2280	flagellar biosynthesis switch protein			-3.88	
XC_2281	RNA polymerase sigma factor			-3.42	
XC_2282	chemotaxis protein			-3.06	
XC_2283	chemotaxis related protein			-3.12	
XC_2284	chemotaxis related protein			-2.66	
XC_2290	hypothetical protein	-6.45	-6.58		
XC_2294	arsenite efflux pump ACR3	-1.79769e+308	INF		
XC_2295	high-affinity Fe2+/Pb2+ permease	-10.60	-10.74		
XC_2298	flagellar motor protein MotD	3.78	3.80		2.24
XC_2299	chromosome partioning protein	2.43		-3.17	
XC_2300	chemotaxis protein	2.55		-2.64	
XC_2301	hypothetical protein	2.56		-3.13	

XC_2302	chemotaxis response regulator	2.83		-2.69	
XC_2303	chemotaxis protein	3.09		-2.62	
XC_2304	chemotaxis protein	2.63			
XC_2305	hypothetical protein	2.55		-2.01	2.43
XC_2306	chemotaxis protein	3.92	3.98	-2.12	2.60
XC_2307	chemotaxis protein	4.31	4.35		
XC_2308	chemotaxis protein	2.64			
XC_2309	chemotaxis protein	4.58	4.64		
XC_2311	chemotaxis protein	3.21		-3.06	
XC_2313	chemotaxis protein	3.77	3.85		
XC_2314	chemotaxis protein	2.85			
XC_2315	methyl-accepting chemotaxis protein	2.34			
XC_2316	chemotaxis protein	2.78			
XC_2318	chemotaxis protein	3.95	3.72	-2.90	
XC_2319	hypothetical protein	3.49	3.62	-3.34	
XC_2320	chemotaxis protein	4.35	4.40	-3.70	
XC_2321	chemotaxis protein methyltransferase	3.22	3.31	-2.94	
XC_2323	chemotaxis-specific methylesterase	2.93			
XC_2342	regulatory protein rpfI	3.12			
XC_2390	tryptophan-rich sensory protein	-4.06			
XC_2393	hypothetical protein		INF		
XC_2394	IS1477 transposase	3.45	3.40		
XC_2396	filamentous hemagglutinin	-11.20	-4.83		
XC_2399	integrase/recombinase	-9.38	-9.51		
XC_2400	DNA helicase-like protein	-10.37	-10.42		
XC_2401	hypothetical protein	-11.23	-10.74		
XC_2402	plasmid mobilization protein		INF		

XC_2403	MchC protein	-10.53	-10.72	
XC_2404	hemolysin activation protein		INF	
XC_2405	transport transmembrane protein		INF	
XC_2406	hypothetical protein	-7.34	-7.38	
XC_2407	hypothetical protein	-10.59	-10.01	
XC_2408	hydroxyproline-rich glycoprotein DZ-HRGP	-10.85	-10.47	
XC_2409	hypothetical protein	-8.61	-8.47	
XC_2410	hypothetical protein	-10.39	-10.56	
XC_2411	hypothetical protein	-6.11	-6.15	
XC_2412	hypothetical protein	-9.51	-9.71	
XC_2413	NTPase VagA	-4.65	-4.74	
XC_2414	hypothetical protein	-10.01	-10.04	
XC_2415	hypothetical protein	-10.26	-10.05	
XC_2416	hypothetical protein	-10.15	-9.85	
XC_2417	plasmid mobilization protein	-10.20	-10.15	
XC_2418	hypothetical protein	-12.31	-12.20	-4.84
XC_2419	hypothetical protein	-10.61	-10.86	-2.69
XC_2420	hypothetical protein	-11.63	-11.96	
XC_2421	phage-related integrase	-10.19	-10.17	
XC_2422	hypothetical protein	-10.59	-10.67	
XC_2423	hypothetical protein	-10.85	-11.03	
XC_2424	hypothetical protein	-11.37	-11.40	-2.35
XC_2425	hypothetical protein	-9.97	-10.58	
XC_2426	RNA-directed DNA polymerase	-10.10	-10.49	
XC_2427	hypothetical protein	-1.79769e+308	INF	
XC_2428	ankyrin repeat-containing protein	-10.31	-10.52	
XC_2429	hypothetical protein	-10.66	-10.81	

XC_2430	transcriptional regulator	-9.06	-9.02	
XC_2431	hypothetical protein	-9.93	-9.98	
XC_2432	hypothetical protein	-11.23	-10.68	
XC_2433	ATPase	-10.17	-10.02	
XC_2434	phage associated protein	-8.87	-9.12	
XC_2435	hypothetical protein	-9.26	-9.51	
XC_2436	hypothetical protein	-8.81	-8.95	
XC_2437	hypothetical protein	-10.01	-10.35	-2.07
XC_2438	plasmid-like protein	-11.06	-10.76	-2.87
XC_2439	hypothetical protein		INF	
XC_2440	hypothetical protein	-8.05	-8.29	
XC_2441	hypothetical protein	-8.75	-8.89	
XC_2442	hypothetical protein	-9.11	-9.27	
XC_2443	hypothetical protein	-9.48	-9.32	
XC_2444	hypothetical protein	-9.71	-9.81	
XC_2456	two-component system sensor protein	3.08		2.47
XC_2458	mannan endo-1,4-beta-mannosidase			-4.27
XC_2459	hypothetical protein	2.31		
XC_2474	hypothetical protein	-10.78	-6.45	
XC_2475	sensor kinase	-10.12	-5.10	
XC_2504	chemotaxis protein	2.83		
XC_2589	phage-related integrase	-7.58	-7.82	
XC_2591	hypothetical protein	-5.03		
XC_2592	RhsD protein	-5.13	-5.19	
XC_2596	hypothetical protein	-9.86	-5.95	
XC_2599	hypothetical protein	-1.79769e+308	INF	
XC_2600	hypothetical protein	-10.20	-10.39	

XC_2601	invertase/recombinase protein	-10.38	-10.54	
XC_2602	avirulence protein	-9.61	-9.89	
XC_2605	hypothetical protein	-3.61	-3.91	
XC_2606	hypothetical protein	-4.47	-4.50	
XC_2607	hypothetical protein	-1.79769e+308	-11.15	
XC_2608	hypothetical protein	-10.64	-10.32	
XC_2609	hypothetical protein	-9.61	-9.76	
XC_2610	hypothetical protein	-10.35	-10.60	
XC_2611	hypothetical protein	-11.11	-11.18	
XC_2614	hypothetical protein	2.40		
XC_2619	phage-related integrase	-10.31	-10.47	
XC_2620	hypothetical protein	-1.79769e+308	INF	
XC_2623	IS1477 transposase	3.43	3.36	
XC_2627	hypothetical protein		INF	
XC_2630	hypothetical protein	-11.24	-11.38	
XC_2631	hypothetical protein	-10.91	-10.58	
XC_2632	hypothetical protein	-7.91	-7.90	
XC_2633	hypothetical protein	-6.15	-6.48	
XC_2634	hypothetical protein	-10.01	-10.23	
XC_2635	hypothetical protein	-9.38	-9.61	
XC_2636	plasmid-like protein	-10.55	-10.62	
XC_2637	hydroxylase	-11.15	-11.32	
XC_2638	phage-related integrase	-5.57	-4.86	
XC_2703	sulfate transporter	-2.67		
XC_2704	carbonic anhydrase	-2.65		
XC_2708	phosphate ABC transporter substrate-binding protein			2.40
XC_2717	hypothetical protein	-4.08	-4.25	

XC_2745	hypothetical protein	-9.85	-10.00	
XC_2746	hypothetical protein	-9.22	-9.40	
XC_2763	molecular chaperone DnaK	2.33		
XC_2774	hypothetical protein	-3.12		
XC_2778	phage-related integrase	-8.67	-8.52	
XC_2781	hypothetical protein	-3.71	-3.84	
XC_2782	hypothetical protein		INF	
XC_2785	helicase	-9.08	-9.17	
XC_2786	hypothetical protein	-8.98	-9.12	
XC_2787	hypothetical protein		INF	
XC_2788	hypothetical protein		INF	
XC_2789	hypothetical protein	-5.27		
XC_2814	hypothetical protein	-2.32		
XC_2830	hypothetical protein	2.65		-3.63
XC_2840	MarR family transcriptional regulator	-2.73		
XC_2848	asparagine synthetase B	-2.55		-2.83
XC_2857	protein U			-5.37
XC_2858	pili assembly chaperone			-3.24
XC_2859	outer membrane usher protein FasD			-2.39
XC_2860	hypothetical protein			-2.36
XC_2921	hypothetical protein	-2.33		
XC_2933	hypothetical protein	-2.74		
XC_2934	RNA polymerase sigma factor	-3.54	-3.41	
XC_2937	hypothetical protein	-2.52		
XC_2938	hypothetical protein	-4.23	-4.07	
XC_2948	hypothetical protein			-2.22
XC_2977	3-hydroxyisobutyrate dehydrogenase			-2.29

XC_2978	enoyl-CoA hydratase			-2.15
XC_2979	enoyl-CoA hydratase			-3.10
XC_2980	acyl-CoA dehydrogenase			-3.46
XC_2981	methylmalonate-semialdehyde dehydrogenase			-3.77
XC_2992	hypothetical protein	-3.57	-4.54	
XC_3035	DNA mismatch repair protein MutS	2.63		
XC_3038	heat shock protein 15-like protein	2.37		
XC_3100	hypothetical protein	-3.09		
XC_3105	IS1478 transposase		INF	
XC_3153	tetracenomycin polyketide synthesis protein	-4.04	-4.24	
XC_3186	hypothetical protein	-4.90	-5.03	
XC_3213	aconitate hydratase	3.53	3.57	
XC_3214	methylcitrate synthase	3.56	3.60	-3.45
XC_3215	2-methylisocitrate lyase			-3.61
XC_3218	hypothetical protein	-3.70		
XC_3219	hypothetical protein	-9.80	-9.95	
XC_3220	hypothetical protein	-10.68	-10.06	
XC_3280	peptidyl-Asp metalloendopeptidase			-2.64
XC_3305	hypothetical protein	-2.40		
XC_3376	extracellular protease	2.30		
XC_3419	MarR family transcriptional regulator	2.29		
XC_3422	hypothetical protein	2.32		
XC_3487	alpha-amylase	-2.47		-3.84
XC_3540	hypothetical protein	2.95		
XC_3549	hypothetical protein	3.82	3.79	
XC_3550	serine protease	2.77		
XC_3553	hypothetical protein			4.09

XC_3554	hypothetical protein			4.15
XC_3555	hypothetical protein			3.53
XC_3556	hypothetical protein			4.35
XC_3582	hypothetical protein	-9.51	-4.26	
XC_3589	hypothetical protein	-2.67		
XC_3591	pectate lyase			-3.84
XC_3619	acetyl transferase/isomerase	-3.73		
XC_3620	sugar translocase	-10.42	-10.77	
XC_3621	hypothetical protein	-10.24	-10.24	
XC_3622	hypothetical protein		INF	
XC_3626	UDP-glucose 4-epimerase	-10.57	-10.32	
XC_3627	GDP-mannose 4,6-dehydratase	-8.23	-8.27	
XC_3628	hypothetical protein	-10.60	-10.62	
XC_3629	hypothetical protein	-10.22	-10.23	
XC_3630	glycosyltransferase	-8.24	-8.29	
XC_3631	kinase	-9.69	-9.78	
XC_3632	ABC transporter ATP binding protein	-10.69	-10.62	
XC_3633	membrane subunit of LPS efflux transporter	-2.81		
XC_3661	hypothetical protein	2.87		
XC_3752	hypothetical protein	-3.63		
XC_3762	cyanide insensitive terminal oxidase	-2.32		-2.64
XC_3763	cyanide insensitive terminal oxidase			-2.30
XC_3766	oxidoreductase	2.86		
XC_3790	transcriptional regulator	3.61	3.63	
XC_3798	hypothetical protein		INF	
XC_3799	transcriptional regulator		INF	
XC_3800	response regulator		-9.95	

XC_3801	histidine kinase/response regulator hybrid protein		-9.90	
XC_3802	avirulence protein		-9.37	
XC_3805	hypothetical protein	-2.44		
XC_3880	hypothetical protein	-10.16		
XC_3881	cationic amino acid transporter	-9.72		
XC_3882	hypothetical protein	-9.53		
XC_3885	dehydrogenase			-3.51
XC_3951	glucosyltransferase	-2.42		
XC_3970	hypothetical protein	-2.23		
XC_3974	hypothetical protein	-2.72		
XC_3975	hypothetical protein	-2.87		
XC_4007	hypothetical protein	-10.41	-10.44	
XC_4008	hypothetical protein	-9.88	-9.81	
XC_4012	hypothetical protein	-2.27		
XC_4013	entericidin A	-2.46		
XC_4034	hypothetical protein			-2.30
XC_4035	hypothetical protein			-2.61
XC_4063	hypothetical protein	-4.29	-4.27	
XC_4121	4-oxalomesaconate hydratase	3.27		
XC_4143	hypothetical protein	-8.07	-5.33	
XC_4144	peptidyl-prolyl cis-trans isomerase		INF	
XC_4152	cytochrome C biogenesis protein	-4.03	-3.79	-5.06
XC_4153	hypothetical protein	-3.64	-3.60	-6.88
XC_4233	ISxac3 transposase	-5.86		
XC_4234	XmnI methyltransferase	-8.35	-8.46	
XC_4261	transcriptional regulator		INF	
XC_4262	short chain dehydrogenase	-10.02	-9.99	

XC_4286	virulence associated protein	-9.47	-8.97	
XC_4291	microcystin dependent protein			-2.77
XC_4292	microcystin dependent protein			-2.20
XC_4293	microcystin dependent protein			-3.57
XC_4294	acetyltransferase			-2.13
XC_4322	hypothetical protein	2.27		

a: Annotation according to Qian et al., (2005).

b: Predicated function based on best BLAST hits searching the bacterial genome database.

c: Fold change in gene expression in selected mutant compared to wild-type strain - log2 scaled fold change of gene expression

Table S5. Genes divergently regulated by different Rpf proteins. Significantly differentially expressed genes were determined by Cufflinks, after Benjamini-Hochberg correction. The fold change is the ratio of mutant FPKM to wild-type FPKM. The genes were ranked on their fold change.

Gene locus[a]	Function[b]	RpfF[c]	RpfC[c]	RpfG[c]	RpfH[c]
XC_1004	TonB-dependent receptor		up	down	
XC_2223	chemotaxis protein		up	down	
XC_2230	hypothetical protein		up	down	
XC_2231	flagellar protein		up	down	
XC_2245	flagellin		up	down	
XC_2299	chromosome partioning protein		up	down	
XC_2300	chemotaxis protein		up	down	
XC_2301	hypothetical protein		up	down	
XC_2302	chemotaxis response regulator		up	down	
XC_2303	chemotaxis protein		up	down	
XC_2305	hypothetical protein		up	down	up
XC_2306	chemotaxis protein	up	up	down	up
XC_2311	chemotaxis protein		up	down	
XC_2318	chemotaxis protein	up	up	down	
XC_2319	hypothetical protein	up	up	down	
XC_2320	chemotaxis protein	up	up	down	
XC_2321	chemotaxis protein methyltransferase	up	up	down	
XC_2830	hypothetical protein		up	down	
XC_3214	methylcitrate synthase	up	up	down	

a: Annotation according to Qian et al., (2005)
b: Predicted function based on best BLAST hits searching the bacterial genome database.
c: Change in gene expression in selected mutant compared to wild-type strain.
(For specific fold change consult Table S5).

Table S6. Quantitative RT-PCR validation of data generated by RNA-Seq. The Table shows fold changes in *rpfF*, *rpfC* and *rpfG* mutants compared to wild-type for a selection of genes as measured by qRT-PCR and RNA-Seq.

Gene locus[a]	Predicted function[b]	RNA-Seq[c]	qRT-PCR[d]	RNA-Seq[e]	qRT-PCR[f]
XC_0063	regulatory protein cII	-10.72	-7	n.d.[g]	n.d.
XC_0065	hypothetical protein	-10.81	-5	n.d.	n.d.
XC_0113	two-component system sensor protein	-10.57	-5	n.d.	n.d.
XC_0128	hypothetical protein	-11.17	-6	n.d.	n.d.
XC_0136	RhsD protein	-11.20	-7	n.d.	n.d.
XC_0223	hypothetical protein	-10.19	-5	n.d.	n.d.
XC_0225	hypothetical protein	-10.53	-5	n.d.	n.d.
XC_0350	hypothetical protein	-10.84	-12	n.d.	n.d.
XC_0351	hypothetical protein	-10.30	-5	n.d.	n.d.
XC_0479	DNA-binding protein	-11.12	-6	n.d.	n.d.
XC_0480	type I site-specific deoxyribonuclease	-10.04	-5	n.d.	n.d.
XC_0856	hypothetical protein	-10.53	-23	n.d.	n.d.
XC_0864	VirB6 protein	-10.54	-5	n.d.	n.d.
XC_0866	hypothetical protein	-10.05	-7	n.d.	n.d.
XC_0867	hypothetical protein	-10.20	-11	n.d.	n.d.
XC_0870	hypothetical protein	-10.96	-5	n.d.	n.d.
XC_0916	BlaI family transcriptional regulator	-10.92	-5	n.d.	n.d.
XC_0922	reductase	-10.87	-14	n.d.	n.d.
XC_1018	phage-related integrase	-11.26	-6	n.d.	n.d.
XC_1019	hypothetical protein	-12.68	-8	n.d.	n.d.
XC_1023	hypothetical protein	-10.20	-3	n.d.	n.d.
XC_1037	hypothetical protein	-10.35	-4	n.d.	n.d.
XC_1039	hypothetical protein	-10.92	-5	n.d.	n.d.
XC_1058	pilin	-11.12	-6	n.d.	n.d.
XC_1381	hypothetical protein	-11.31	-7	n.d.	n.d.
XC_1624	PilX protein	-10.72	-5	n.d.	n.d.

XC_1625	PilY1 protein	-10.15	-5	n.d.	n.d.
XC_1626	type IV pilin	-10.47	-5	n.d.	n.d.
XC_1633	VirB9 protein	-10.26	-11	n.d.	n.d.
XC_1634	VirB10 protein	-10.42	-12	n.d.	n.d.
XC_1637	VirB2 protein	-10.31	-5	n.d.	n.d.
XC_1638	VirB3 protein	-10.45	-2	n.d.	n.d.
XC_2013	sensor kinase	-10.49	-4	n.d.	n.d.
XC_2016	VirB6 protein	-10.61	-5	n.d.	n.d.
XC_2059	hypothetical protein	-10.82	-19	n.d.	n.d.
XC_2060	hypothetical protein	-10.41	-5	n.d.	n.d.
XC_2400	DNA helicase-like protein	n.d.	n.d.	-10.42	-3
XC_2401	hypothetical protein	n.d.	n.d.	-10.74	-14
XC_2402	plasmid mobilization protein	n.d.	n.d.	-23.00	-5
XC_2403	MchC protein	n.d.	n.d.	-10.72	-5
XC_2404	hemolysin activation protein	n.d.	n.d.	-42.00	-12
XC_2405	transport transmembrane protein	n.d.	n.d.	-11.00	-5
XC_2406	hypothetical protein	n.d.	n.d.	-7.38	-6
XC_2407	hypothetical protein	n.d.	n.d.	-10.01	-2
XC_2408	hydroxyproline-rich glycoprotein DZ-HRGP	n.d.	n.d.	-10.47	-11
XC_2409	hypothetical protein	n.d.	n.d.	-8.47	-7
XC_2410	hypothetical protein	n.d.	n.d.	-10.56	-9
XC_2411	hypothetical protein	n.d.	n.d.	-6.15	-5
XC_2412	hypothetical protein	n.d.	n.d.	-9.71	-23
XC_2413	NTPase VagA	n.d.	n.d.	-4.74	-3
XC_2414	hypothetical protein	n.d.	n.d.	-10.04	-5
XC_2415	hypothetical protein	n.d.	n.d.	-10.05	-5
XC_2416	hypothetical protein	n.d.	n.d.	-9.85	-7
XC_2417	plasmid mobilization protein	n.d.	n.d.	-10.15	-7

a: Annotation according to Qian et al., (2005)
b: Predicated function based on best BLAST hits searching the bacterial genome database.

c: Fold change in gene expression in *rpfC* mutant compared to wild-type as measured by RNA-Seq
d: Fold change in gene expression in *rpfC* mutant compared to wild-type as measured by qRT-PCR
e: Fold change in gene expression in *rpfG* mutant compared to wild-type as measured by RNA-Seq
f: Fold change in gene expression in *rpfG* mutant compared to wild-type as measured by qRT-PCR
g: Not done (n.d.)

Table S7. The effects of mutation of previously annotated genes in *Xcc* identified to be regulated by RpfF, RpfC and/or RpfG on virulence. The virulence of each mutant was tested by measurement of the lesion length after bacteria were introduced into the vascular system of Chinese radish by leaf clipping.

Gene locus[a]	Predicted Function[b]	Regulation[c]	Virulence[d]
XC_0027	cellulase	RpfG	III
XC_0094	tldD protein	RpfC	III
XC_0113	two-component system sensor protein	RpfF, RpfC	III
XC_0130	hypothetical protein	RpfF	III
XC_0224	methyltransferase	RpfF, RpfC	III
XC_0225	hypothetical protein	RpfF, RpfC	III
XC_0323	transcriptional regulator	RpfF	III
XC_0479	DNA-binding protein	RpfF, RpfC	III
XC_0480	type I site-specific deoxyribonuclease	RpfF, RpfC	III
XC_0586	hypothetical protein	RpfF, RpfC	III
XC_0637	histidine kinase/response regulator hybrid protein	RpfC	III
XC_0710	hypothetical protein	RpfF, RpfC	II
XC_0865	hypothetical protein	RpfF, RpfC	III
XC_1036	hypothetical protein	RpfF	III
XC_1037	hypothetical protein	RpfF, RpfC	III
XC_1038	hypothetical protein	RpfF	II
XC_1154	membrane transport protein	RpfC	I
XC_1624	PilX protein	RpfF, RpfC	III
XC_1660	GumD protein	RpfC	II
XC_1853	hypothetical protein	RpfC	III
XC_2055	hypothetical protein	RpfF, RpfC	III
XC_2234	flagellar basal body rod protein FlgB	RpfG	II
XC_2237	flagellar hook protein FlgE	RpfG	III

XC_2241	flagellar basal body P-ring protein	RpfG	III
XC_2260	flagellar MS-ring protein	RpfG	III
XC_2277	flagellar biosynthesis protein FlhB	RpfG	III
XC_2280	flagellar biosynthesis switch protein	RpfG	III
XC_2298	flagellar motor protein MotD	RpfF, RpfC	II
XC_2313	chemotaxis protein	RpfF, RpfC	I
XC_2316	chemotaxis protein	RpfC	III
XC_2948	hypothetical protein	RpfG	III
XC_2977	3-hydroxyisobutirate dehydrogenase	RpfG	III
XC_2981	methylmalonate-semialdehyde dehydrogenase	RpfG	III
XC_3213	aconitate hydratase	RpfF, RpfC	III
XC_3554	membrane protein	RpfG	III
XC_3555	glycosyltransferase	RpfG	III
XC_3620	sugar translocase	RpfF, RpfC	III
XC_3626	UDP-glucose 4-epimerase	RpfF, RpfC	III
XC_3627	GDP-mannose 4,6-dehydratase	RpfF, RpfC	III
XC_3628	hypothetical protein	RpfF, RpfC	III
XC_3629	hypothetical protein	RpfF, RpfC	III
XC_3630	glycosyltransferase	RpfF, RpfC	II
XC_3631	kinase	RpfF, RpfC	I
XC_3632	ABC transporter ATP binding protein	RpfF, RpfC	III
XC_3633	membrane subunit of LPS efflux transporter	RpfC	III
XC_3800	response regulator	RpfF	III
XC_4143	hypothetical protein	RpfF, RpfC	III
XC_4153	hypothetical protein	RpfF, RpfC, RpfG	III
XC_4293	microcystin dependent protein	RpfG	III

a:Designation of the gene locus that was inactivated by mutation (annotation according to Qian et al (2005)).
b: Predicated function based on best BLAST hits searching the bacterial genome database.
c: The element of the Rpf/DSF system that regulated this gene - specfic fold change of gene expression is detailed in Table S3.
d: Virulence phenotypes in Chinese Radish as a consequence of mutation of genes as adapted from classification defined in Figure 3.

Table S8. Effects of combinatorial mutation of previously annotated genes on the virulence of *Xcc* to Chinese radish. The virulence of each mutant was tested by measurement of the lesion length after bacteria were introduced into the vascular system of Chinese radish by leaf clipping. Double mutations did not give rise to an additive effect on virulence.

Genes that were inactivated[a]	Virulence[b]
XC_0710\XC_0027	II
XC_1038\XC_0027	II
XC_1660\XC_0027	II
XC_2234\XC_0027	II
XC_2298\XC_0027	II
XC_3630\XC_0027	II
XC_0710\XC_0323	II
XC_1038\XC_0323	II
XC_1660\XC_0323	II
XC_2234\XC_0323	II
XC_2298\XC_0323	II
XC_3630\XC_0323	II
XC_0710\XC_0480	II
XC_1038\XC_0480	II
XC_1660\XC_0480	II
XC_2234\XC_0480	II
XC_2298\XC_0480	II
XC_3630\XC_0480	II
XC_0710\XC_0637	II
XC_1038\XC_0637	II
XC_1660\XC_0637	II
XC_2234\XC_0637	II
XC_2298\XC_0637	II
XC_3630\XC_0637	II

a: Designation of the gene locus that was inactivated by mutation (annotation according to Qian et al [2005]).
b: Virulence phenotypes in Chinese Radish as a consequence of mutation of genes as adapted from classification defined in Figure 3.

Table S9. List of the 68 unannotated genes regulated by RpfF, RpfC and/or RpfG and the influence of mutation of these genes on virulence of *Xcc* to Chinese radish.

Transcript designation[a]	Start[b]	End[b]	Length (nt)	Insertion site[c]	Virulence[d]
TID2	50022	50402	381	50344	I
TID3	77017	77220	204	77056	V
TID5	78431	79402	972	78435	V
TID6	80767	81213	447	80792	II
TID8	163742	164440	699	164204	V
TID9	164621	164956	336	164871	V
TID10	171586	172836	1251	171623	V
TID16	394150	394791	642	394210	V
TID17	404902	405264	363	405244	V
TID18	407169	407657	489	407265	V
TID26	550132	551613	1482	550467	V
TID28	577673	577957	285	577887	IV
TID29	601193	601891	699	601210	V
TID30	686074	686502	429	686411	V
TID32	710291	710434	144	710394	IV
TID36	838746	839714	969	839341	I
TID45	1040519	1041289	771	1040535	V
TID46	1059945	1060547	603	1060466	V
TID47	1089233	1089613	381	1089287	V
TID49	1161375	1161761	387	1161416	V
TID51	1233244	1233663	420	1233610	V
TID53	1246507	1246767	261	1246617	V
TID56	1248791	1249162	372	1249055	V
TID58	1254044	1254403	360	1254156	V
TID60	1265318	1265524	207	1265375	II
TID61	1388066	1388383	318	1388148	V

TID63	1468983	1469114	132	1468994	V
TID64	1568785	1569600	816	1569031	II
TID68	1744147	1744494	348	1744382	I
TID75	2193981	2194241	261	2194226	IV
TID81	2422070	2422750	681	2422490	V
TID82	2467081	2467329	249	2467118	V
TID86	2486779	2487129	351	2486784	V
TID88	2489339	2490940	1602	2489356	V
TID89	2531915	2532082	168	2531946	V
TID91	2536676	2536984	309	2536962	V
TID93	2540172	2540459	288	2540200	V
TID97	2566469	2566744	276	2566512	V
TID98	2573230	2573448	219	2573378	V
TID99	2584403	2584846	444	2584427	V
TID100	2611087	2611344	258	2611124	IV
TID101	2764514	2764882	369	2764633	V
TID102	2768924	2769202	279	2769003	V
TID107	2947726	2947938	213	2947781	V
TID181	3007215	3007325	111	3007286	V
TID114	3130655	3130939	285	3130842	V
TID116	3131695	3132516	822	3132250	V
TID118	3140515	3140955	441	3140731	II
TID119	3142126	3142941	816	3142560	V
TID120	3148912	3149193	282	3148925	I
TID121	3159345	3160106	762	3159529	V
TID122	3163929	3164765	837	3164299	V
TID123	3170824	3171354	531	3171119	V
TID130	3519895	3520152	258	3519965	V
TID132	3592803	3593480	678	3593001	V
TID135	3627629	3627952	324	3627935	II
TID136	3630004	3630723	720	3630141	V

TID143	3773215	3773388	174	3773293	V
TID148	4063928	4064359	432	4064170	V
TID151	4214753	4215184	432	4214803	V
TID152	4265199	4265864	666	4265229	V
TID153	4305065	4305484	420	4305100	IV
TID156	4413491	4414270	780	4413537	III
TID158	4568882	4569238	357	4568883	V
TID160	4624524	4625456	933	4624622	V
TID166	4862456	4863091	636	4862690	V
TID176	5066234	5066443	210	5066288	V
TID177	5068515	5068715	201	5068575	II

a: Designation of novel transcripts
b: Annotation taken from Qian et al., [2005].
c: Insertion site for Tn5 antibiotic resistance cassette.
d: Virulence phenotypes in Chinese Radish as a consequence of mutation of genes as adapted from classification defined in Figure 3.

Table S10. Table of strains and plasmids used in this study.

**Strain or plasmid**	**Relevant characteristics**	**Source or Reference**
Xcc 8004	Rif^R	Qian et al., (2005)
Xcc 8005/ pPH1JI	Gm^R	Qian et al., (2005)
pRK2073	Helper plasmid, Sp^R	Leong et al., (1982)
pK18*mobkan*	*lacZa*, Km^R	Schäfer et al., (1994)
pK18*mobtet*	*lacZa*, Tc^R	Tang et al., (2005)
pK18*mobsacB*	*sacB*, *lacZa*, KmR allelic exchange vector	Schäfer et al., (1994)
pLAFR1::Tn5gusA5	Broad-host-range IncP2, cosmid, Km^R, Tc^R	Ryan et al., (2007)
pLAFR3	Broad-host-range IncP2, cosmid, Tc^R	Staskawicz et al., (1984)
pPH1JI	Sp^R, Gm^R	Turner et al., (1985)

**Mutants created with pK18*mobkan***	**All Rif^R, Km^R**	
XC0027	As 8004, but *XC_0027*::pK18*mob*(polar)	This study
XC1036	As 8004, but *XC_1036*::pK18*mob*(polar)	This study
XC3633	As 8004, but *XC_3633*::pK18*mob*(polar)	This study
XC4153	As 8004, but *XC_4153*::pK18*mob*(non-polar)	This study
**Transposon-insertion mutants**	**All Rif^R, Km^R, Sp^R, Gm^R**	
XC0094	As 8004, but *XC_0094*::Tn5gusA5	This study
XC0113	As 8004, but *XC_0113*::Tn5gusA5	This study
XC0130	As 8004, but *XC_0130*::Tn5gusA5	This study
XC0224	As 8004, but *XC_0224*::Tn5gusA5	This study
XC0225	As 8004, but *XC_0225*::Tn5gusA5	This study
XC0323	As 8004, but *XC_0323*::Tn5gusA5	This study
XC0479	As 8004, but *XC_0479*::Tn5gusA5	This study

XC0480	As 8004, but *XC_0480*::Tn5gusA5	This study
XC0586	As 8004, but *XC_0586*::Tn5gusA5	This study
XC0637	As 8004, but *XC_0637*::Tn5gusA5	This study
XC0710	As 8004, but *XC_0710*::Tn5gusA5	This study
XC0865	As 8004, but *XC_0865*::Tn5gusA5	This study
XC1037	As 8004, but *XC_1037*::Tn5gusA5	This study
XC1038	As 8004, but *XC_1038*::Tn5gusA5	This study
XC1154	As 8004, but *XC_1154*::Tn5gusA5	This study
XC1624	As 8004, but *XC_1624*::Tn5gusA5	This study
XC1660	As 8004, but *XC_1660*::Tn5gusA5	This study
XC1853	As 8004, but *XC_1853*::Tn5gusA5	This study
XC2055	As 8004, but *XC_2055*::Tn5gusA5	This study
XC2234	As 8004, but *XC_2234*::Tn5gusA5	This study
XC2237	As 8004, but *XC_2237*::Tn5gusA5	This study
XC2241	As 8004, but *XC_2241*::Tn5gusA5	This study
XC2260	As 8004, but *XC_2260*::Tn5gusA5	This study
XC2277	As 8004, but *XC_2277*::Tn5gusA5	This study
XC2280	As 8004, but *XC_2280*::Tn5gusA5	This study

XC2298	As 8004, but *XC_2298*::Tn5gusA5	This study
XC2313	As 8004, but *XC_2313*::Tn5gusA5	This study
XC2316	As 8004, but *XC_2316*::Tn5gusA5	This study
XC2948	As 8004, but *XC_2948*::Tn5gusA5	This study
XC2977	As 8004, but *XC_2977*::Tn5gusA5	This study
XC2981	As 8004, but *XC_2981*::Tn5gusA5	This study
XC3213	As 8004, but *XC_3213*::Tn5gusA5	This study
XC3554	As 8004, but *XC_3554*::Tn5gusA5	This study
XC3555	As 8004, but *XC_3555*::Tn5gusA5	This study
**Double mutants with Tn*5gusA*5 and pK18*mobtet***	**All Rif^R, Km^R, Sp^R, Tc^R**	
XC_0710\XC_0027	*XC_0027*::pK18*mob*, *XC_0710*::Tc	This study
XC_1038\XC_0027	*XC_0027*::pK18*mob*, *XC_1038*::Tc	This study
XC_1660\XC_0027	*XC_0027*::pK18*mob, XC_1660*::Tc	This study
XC_2234\XC_0027	*XC_0027*::pK18*mob, XC_2234*::Tc	This study
XC_2298\XC_0027	*XC_0027*::pK18*mob, XC_2298*::Tc	This study
XC_3630\XC_0027	*XC_0027*::pK18*mob, XC_3630*::Tc	This study

XC_0710\XC_0323	*XC_0323*::Tn5gusA5, *XC_0710*::Tc	This study
XC_1038\XC_0323	*XC_0323*::Tn5gusA5, *XC_1038*::Tc	This study
XC_1660\XC_0323	*XC_0323*::Tn5gusA5, *XC_1660*::Tc	This study
XC_2234\XC_0323	*XC_0323*::Tn5gusA5, *XC_2234*::Tc	This study
XC_2298\XC_0323	*XC_0323*::Tn5gusA5, *XC_2298*::Tc	This study
XC_3630\XC_0323	*XC_0323*::Tn5gusA5, *XC_3630*::Tc	This study
XC_0710\XC_0480	*XC_0480*::Tn5gusA5, *XC_0710*::Tc	This study
XC_1038\XC_0480	*XC_0480*::Tn5gusA5, *XC_1038*::Tc	This study
XC_1660\XC_0480	*XC_0480*::Tn5gusA5, *XC_1660*::Tc	This study
XC_2234\XC_0480	*XC_0480*::Tn5gusA5, *XC_2234*::Tc	This study
XC_2298\XC_0480	*XC_0480*::Tn5gusA5, *XC_2298*::Tc	This study
XC_3630\XC_0480	*XC_0480*::Tn5gusA5, *XC_3630*::Tc	This study
XC_0710\XC_0637	*XC_0637*::Tn5gusA5, *XC_0710*::Tc	This study
XC_1038\XC_0637	*XC_0637*::Tn5gusA5, *XC_1038*::Tc	This study
XC_1660\XC_0637	*XC_0637*::Tn5gusA5, *XC_1660*::Tc	This study
XC_2234\XC_0637	*XC_0637*::Tn5gusA5, *XC_2234*::Tc	This study

XC_2298\XC_0637	*XC_0637*::Tn5gusA5, *XC_2298*::Tc	This study
XC_3630\XC_0637	*XC_0637*::Tn5gusA5, *XC_3630*::Tc	This study

Qian W, Jia YT, Ren SX, He YQ, Feng JX, Lu LF, Sun QH, Ying G, Tang DJ, Tang H, et al. Comparative and functional genomic analyses of the pathogenicity of phytopathogen Xanthomonas campestris pv. campestris. Genome Res. 2005;15:757-767.

Ryan RP, Fouhy Y, Lucey JF, Jiang B-L, He Y-Q, Feng J-X, Tang J-L, Dow JM. Cyclic di-GMP signaling in the virulence and environmental adaptation of Xanthomonas campestris. Mol. Microbiol. 2007;63:429-442.

Leong, S. A., G. S. Ditta, and D. R. Helinski. 1982. Heme biosynthesis in Rhizobium: identification of a cloned gene coding for δ-aminolevulinic acid synthetase from Rhizobium meliloti. J. Biol. Chem. 257:8724-8730.

Schäfer, A., Tauch, A., Jäger, W., Kalinowski, J., Thierbach, G. & Pühler, A. (1994). Small mobilizable multi-purpose cloning vectors derived from the Escherichia coli plasmids pK18 and pK19: selection of defined deletions in the chromosome of Corynebacterium glutamicum. Gene 145, 69–73.

Tang, D.J., Li, X.J., He, Y.Q., Feng, J.X., Chen, B., and Tang, J.L. (2005) The zinc uptake regulator Zur is essential for the full virulence of Xanthomonas campestris pv. campestris. Mol Plant Microbe Interact **18**: 652–658.

Staskawicz B. , Dahlbeck D. , Keen N. , Napoli C. (1987) Molecular characterization of cloned avirulence genes from race 0 and race 1 of Pseudomonas syringae pv. glycinea. J. Bacteriol 169:5789–5794.

Turner, P., C. E. Barber, and M. J. Daniels. 1985. Evidence for clustered pathogenicity genes in Xanthomonas campestris pv. campestris. Mol. Gen. Genet. 199:338
